# A novel unbalanced translocation between the short arms of chromosomes 6 and 16 in a newborn girl: Clinical features and management

**DOI:** 10.1002/ccr3.1574

**Published:** 2018-05-24

**Authors:** Paula de Sousa, Alasdair Kennedy, Heva H. S. Lalani

**Affiliations:** ^1^ Department of Paediatrics East Surrey Hospital Redhill UK; ^2^ Department of Ophthalmology Worthing Hospital Worthing UK; ^3^ Department of Paediatrics Conquest Hospital Hastings UK

**Keywords:** 16p12.3 duplication, 6p25.3 deletion, comparative genomic hybridization (CGH) analysis, genetic, unbalanced translocation

## Abstract

The reporting of previously undescribed genetic mutations and resulting clinical phenotypes guides management and enables a more accurate prognosis for clinicians treating newborns with similar features. Previous cases of 6p deletions and 16p duplications have been described as separate entities. This patient presents with both and has a unique phenotype.

## 1 INTRODUCTION

6p deletions and 16p duplications rarely occur simultaneously. According to the^1^ database, there are 74 reported cases of isolated 6p deletions and 44 cases associated with other genetic abnormalities such as deletions and duplications on another chromosome.^1^

16p duplications are more common with 290 cases occurring in isolation and 82 cases associated with other deletions and duplications.^1^ These abnormalities can either be inherited or occur de novo.

We present a patient with a 6p25.3 deletion and 16p12.3 duplication recognized using array comparative genomic hybridization (CGH) analysis of deoxyribonucleic acid (DNA). This combination has not previously been described.

There have been 32 reports of 6p25 deletions in isolation in the Unique database.^1^ The role of the majority of the genes in the 6p25 region is unclear, but forkhead box C1 (*FOXC1*) found in 6p25.3 plays a vital role in embryogenesis during the development of the anterior structures of the eye and also has an effect on cardiac and brain embryogenesis.^2^

Terminal duplications of 16p that have been reported include 33 reports on 16p13.3 duplications and 100 reports on 16p13.11 duplications and these have been well described.^1^, ^3^

We present the medical features of this novel genetic mutation during the patient's first year of life and compare them with similar genotypes and phenotypes.

## 2 CLINICAL FEAUTRES

An infant girl was born to non‐consanguineous parents. This was their first child, and there was no relevant family history.

An antenatal ultrasound scan at 20 weeks revealed a ventricular septal defect, an overriding aorta, bilateral superior vena cava with a dilated coronary sinus, a hyperechogenic bowel, an enlarged cisterna magna, and bilateral dysmorphic feet. At the time, her parents chose not to undergo further investigation.

She was born at 36 weeks and 2 days weighing 2195 g (9th percentile) with a head circumference of 31 cm (9th percentile) via emergency cesarean section due to evidence of fetal distress on cardiotocography and breech presentation. She was born in moderate condition, with thin meconium and required inflation breaths. Her Apgar scores were 6 and 9 at 1 and 5 min, respectively.

She was admitted to the special care baby unit and was started on continuous positive airway pressure. She was weaned to humidified high‐flow nasal cannulae after 1 day and onto simple nasal oxygen cannulae after 2 days.

On initial examination, she had generalized hypotonia, an erythematous birth mark over her posterior fontanel, microphthalmia, hypertelorism, low set ears, a high arched palate, a cleft of the left soft palate, microstomia, micrognathia, a short neck, a sacral dimple, arthrogryposis, bilateral congenital vertical talus, talipes equinovarus, bilateral calcaneal valgus, single palmar creases, pollex varus, and a widened gap between the second and third digits of the hands.

### 2.1 Cardiac and respiratory

An echocardiogram soon after birth showed a large subarterial ventricular septal defect with a small apical ventricular septal defect, a patent foramen ovale, mild mitral regurgitation, an overriding aorta, a patent ductus arteriosus, and bilateral superior vena cava.

She was discharged after 1 month but readmitted 8 days later with increased work of breathing, desaturation, choking episodes, cyanotic spells, and an episode of supraventricular tachycardia requiring adenosine. She was treated with furosemide, spironolactone, and propranolol. A chest X‐ray showed cardiomegaly, and a cardiac rhythm monitor revealed sinus rhythm with a superior axis with some ventricular ectopics and prominence of right‐sided voltages. She underwent closure of her ventricular septal defect at 2 months of age, and at 4 months of age, she had further cardiac surgery and spent 5 months on the ward, following 7 weeks of invasive ventilation. She had a residual murmur after surgery, which disappeared by 11 months of age. Following this admission, she had a chronic oxygen requirement of 0.2 L via nasal cannulae with baseline subcostal recessions and a tracheal tug.

Additionally, she was diagnosed with pulmonary hypertension, which subsequently resolved by 11 months of age. A computed tomography scan revealed small‐volume lungs with bilateral parenchymal changes. She had a further two separate admissions at 7 months and 10 months of age with viral‐induced wheeze, and at 10 months of age, she was treated with home salbutamol nebulizers and prophylactic azithromycin and received the flu and respiratory syncytial virus vaccines.

### 2.2 Gastroentrology and feeds

She was diagnosed with reflux at 1 month of age and was treated with omeprazole and a preparation containing sodium alginate and magnesium alginate. An abdominal ultrasound was unremarkable. She was initially on 4‐hourly high‐energy formula feeds via a bottle using a MAM teat with nasogastric top‐ups. At 7 months of age, she was reliant on nasogastric feeds due to fatigue secondary to her cardiac issues. There was no plan for a gastrostomy tube as it was felt that once her cardiac issues and cleft palate were treated, she would be able to tolerate oral feeds.

### 2.3 Growth and development

Her height, weight, and head circumference remained below the 0.4th percentile during the first year of life. She initially had poor weight gain, and this was subsequently reversed with dietetic input. She has global developmental delay, and at 7 months of age, she was able to roll from side to side, reach for objects, smile, giggle, and hold her head steady when upright but not able to sit independently.

### 2.4 Neurological

**Figure 1:**
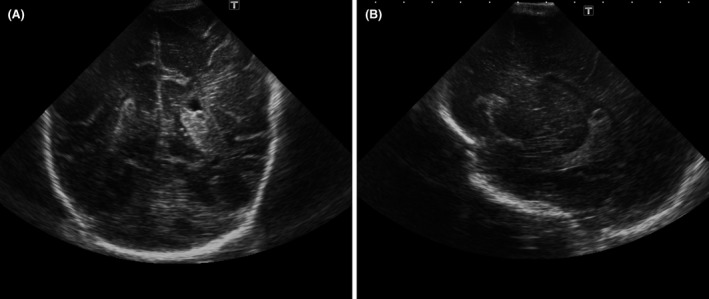
A, Coronal section of a cranial ultrasound of the patient demonstrating a bulky choroid plexus on the left side. B, Sagittal section of a cranial ultrasound of the patient demonstrating a choroid plexus cyst

**Figure 2:**
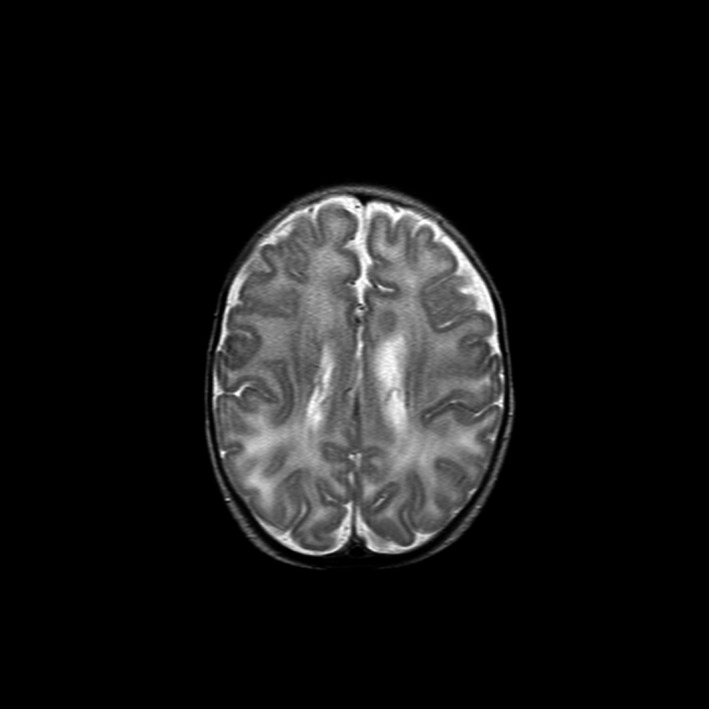
Coronal section of a T2 weighted MRI of the patient's head demonstrating a bulky choroid plexus on the left

There were some concerns regarding seizures in the first year of life, and she had three electroencephalograms that showed possible abnormal unilateral signals, but these seizure‐like episodes have now resolved. Cranial ultrasound revealed a bulky choroid plexus on the left side with a tiny cyst (Figure 1). These changes were noted in the magnetic resonance imaging (MRI) of her head but were of uncertain clinical significance (Figure 2).

### 2.5 Otorhinolaryngology and ophthalmology

Surgery for her left‐sided cleft of the soft palate has been put on hold due to her complex medical background. She had congenital stridor for which she had a bronchoscopy that ruled out laryngomalacia at 4 months of age. This stridor subsequently resolved. Initial hearing assessments confirmed intact cochleae, but subsequent assessments have shown a mild reduction in hearing secondary to an otitis media with an effusion. She has horizontal nystagmus with posterior embryotoxon and ocular albinism, and the ophthalmology team felt that she had some functional vision.

### 2.6 Skeletal

She had a right dislocated hip at birth and was found to have congenital hip dysplasia on the right and acquired hip dysplasia on the left. She has a sacral dimple, arthrogryposis, bilateral congenital vertical talus, talipes equinovarus, bilateral calcaneal valgus, pollex varus, and a widened gap between the second and third digits of the hands. A spinal ultrasound was unremarkable.

## 3 INVESTIGATIONS

**Figure 3:**
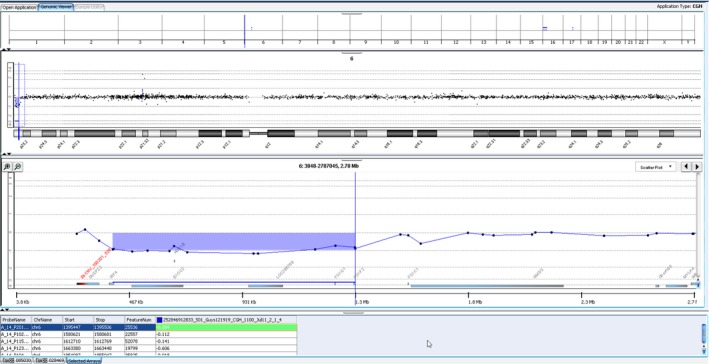
Array CGH trace of the patient's chromosome 6 deletion

**Figure 4:**
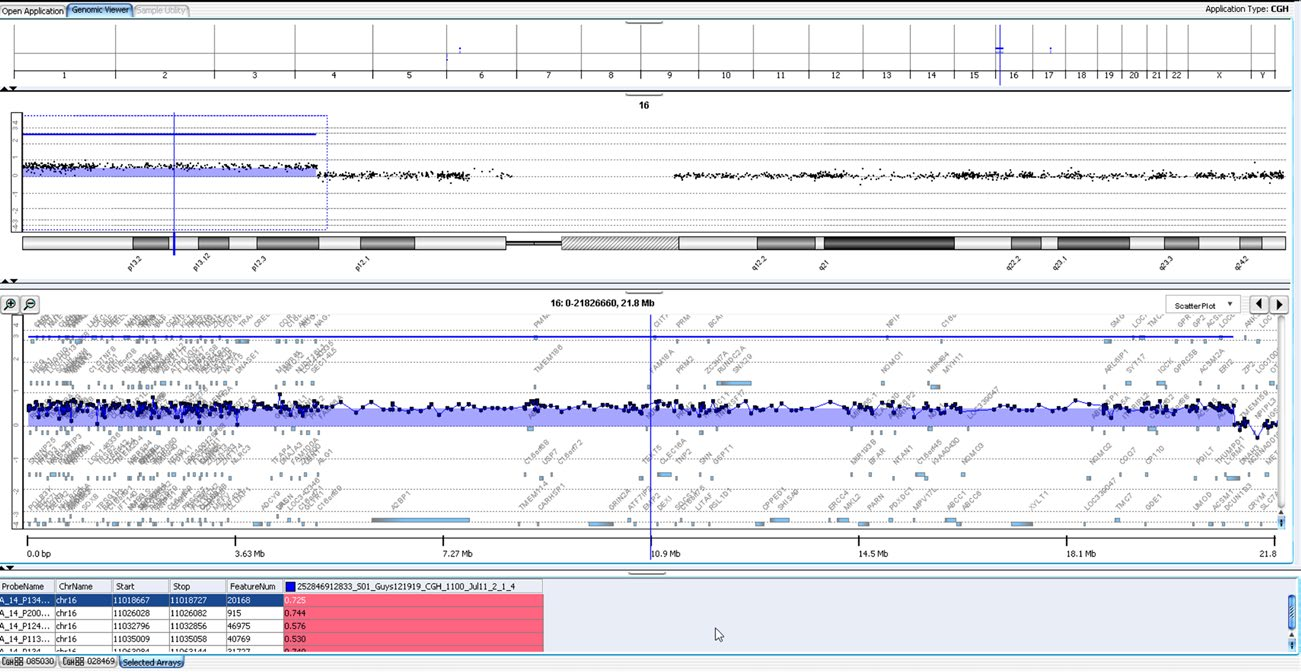
Array CGH trace of the patient's chromosome 16 duplication

Array CGH analysis of this infant was carried out using oligonucleotide arrays with ~60 000 probes across the genome. It identified two areas of imbalance: the first, a terminal deletion of approximately 1.395 Mb from the short arm of chromosome 6, from pter to band p25.3 and the second, a terminal duplication of approximately 21.063 Mb from the short arm of chromosome 16, from pter to band p12.3 (Figures 3 and 4, respectively). These findings are consistent with an unbalanced translocation between the short arms of chromosomes 6 and 16, reported as arr6p25.3 (407 462‐1 395 506) × 1 and 16p13.3p12.3 (96 765‐21 062 537) × 3. Through the use of the University of California, Santa Cruz (UCSC) genome browser,^4^ the genes affected in this mutation are listed in Appendices 1 and S1. The short arm of chromosome 6 deletion included FOXF2, FOXQ1, IRF‐4, EXOC2, HUS1B, LINC01394, and several ribonucleic acid (RNA) regulation genes. The patient's parents have had genetic tests and have declined disclosure of results.

## 4 DISCUSSION

This is the first reported case of an unbalanced translocation between the short arms of chromosomes 6 and 16 in an infant with predominantly ocular, cardiac, respiratory, and dysmorphic sequelae. As previously mentioned, FOXC1 can be implicated in 6p deletions, but it appeared to lie just proximal to our patient's imbalance in the Array CGH trace (Figures 3 and 4). However, FOXQ1 was one of the deleted genes and has been shown to be involved in embryogenesis. It is expressed in the stomach, bladder, trachea, and salivary glands. The relevance of this in the present case is currently unknown.^5^

Isolated 6p deletions and 16p duplications have been well documented in the literature.

Chromosome 6p deletions are rare but can be divided into two distinct syndromes: terminal and interstitial deletions.

In terminal 6p25 deletions, features in common with the patient include developmental delay, facial dysmorphisms such as hypertelorism, high arched palate, oculocraniofacial defects such as cleft lip or palate, anterior segment dysgenesis of the eye such as posterior embryotoxon, single palmar crease, supraventricular tachycardia, cardiac malformations—mainly atrial and ventricular septal defects in combination with a patent ductus arteriosus and patent foramen ovale and skeletal malformations.^1^, ^6^, ^7^, ^8^, ^9^ Other features of this deletion which our patient did not present with include hydrocephalus, dental hypoplasia, hearing loss, renal malformations, involuted periumbilical skin, delayed bone maturation, neuronal defects, learning disabilities, and behavioral problems.^1^, ^6^, ^7^, ^8^, ^9^

Interstitial deletions of 6p24‐6p22 material result in structural eye anomalies, a short neck, cardiac defects, kidney defects, limb defects, and developmental delay. Most 6p deletion features are apparent at birth, implying that these genes are implicated in embryogenesis.^1^, ^9^, ^18^

16p13.3 duplication is often characterized by developmental delay, poor growth, a high arched palate, cleft lip or palate, micrognathia, congenital heart defects including ventricular septal defects, patent ductus arteriosus and pulmonary hypertension, congenital hip dislocation, and seizures. These features were all present in this patient.^1^

Other features reported in 16p13.3 duplication which did not manifest in the patient include the following: thin hair, high forehead, sparse eyebrows, blepharophimosis with palpebral ptosis, strabismus, short nose, open mouth with everted upper lip, wide‐spaced teeth, everted ears, vascular defects, urogenital anomalies, inguinal hernias, terminal hypoplasia of the distal phalanges, proximally implanted thumbs, and severe learning difficulties.^1^, ^10^, ^11^, ^12^, ^13^, ^14^

In 16p13.11 duplications, features shared with the patient include developmental delay, laryngomalacia, and seizures.^15^, ^16^, ^17^ Other features that this infant did not have include congenital heart defects such as pulmonary stenosis, tetralogy of fallot, coarctation of the aorta, transposition of the great arteries and later in life thoracic aortic aneurysm dissections, hypermobile joints, polydactyly, clinodactyly, brachycephaly, umbilical hernia, and pectus excavatum.^15^, ^16^, ^17^

Our patient's phenotype includes elements from all three genetic abnormalities described above but also contains characteristics which are not associated with them. Other features may present later in childhood or adult life, for example learning difficulties. This is a unique genetic abnormality, previously undescribed and of a challenging nature clinically. This report can be used as a reference for clinicians should similar cases arise. The reporting of previously undescribed genetic mutations and the resulting phenotype guides management and enables an informed prognosis for clinicians treating newborns with similar features.

## CONFLICT OF INTEREST

None declared.

## AUTHOR CONTRIBUTIONS

PS: Contributed to the acquisition of data, drafted the manuscript, approved the final version of the manuscript, and agreed to be accountable for all aspects of the work. AK: Contributed to the acquisition of data, drafted the manuscript, approved the final version of the manuscript, and agreed to be accountable for all aspects of the work. HHS: Contributed to the acquisition of data, revised the manuscript, approved the final version of the manuscript, and agreed to be accountable for all aspects of the work.
