# Supplementary material for: A novel unbalanced translocation between the short arms of chromosomes 6 and 16 in a newborn girl: Clinical features and management
Source: Clin Case Rep. 2018 May 24;6(7):1282–6. doi: 10.1002/ccr3.1574 (PMC6028415; doi:10.1002/ccr3.1574)
Supplement: Supplementary file 1 [file CCR3-6-1282-s001.docx]

Table S1. List of genes deleted in chromosome 6 deletion: 6p25.3 (407 462-1 395 506) × 1 generated from USCS genome browser (Human Genome 38).

| chr6 | 391738 | 411447 | IRF4 | Homo sapiens interferon regulatory factor 4 (IRF4), transcript variant 1, mRNA. (from RefSeq NM_002460) |
| --- | --- | --- | --- | --- |
| chr6 | 485132 | 693111 | EXOC2 | Homo sapiens exocyst complex component 2 (EXOC2), transcript variant 1, mRNA. (from RefSeq NM_018303) |
| chr6 | 524170 | 525581 | RP1-20B11.2 | RP1-20B11.2 (from geneSymbol) |
| chr6 | 655938 | 656963 | HUS1B | Homo sapiens HUS1 checkpoint clamp component B (HUS1B), mRNA. (from RefSeq NM_148959) |
| chr6 | 708591 | 711405 | RP11-532F6.3 | Homo sapiens cDNA FLJ35503 fis, clone SMINT2009216. (from mRNA AK092822) |
| chr6 | 711532 | 750729 | RP11-532F6.4 | RP11-532F6.4 (from geneSymbol) |
| chr6 | 761674 | 780648 | RP11-532F6.5 | RP11-532F6.5 (from geneSymbol) |
| chr6 | 774746 | 780214 | RP11-284J1.1 | Homo sapiens cDNA FLJ36084 fis, clone TESTI2020029. (from mRNA AK093403) |
| chr6 | 905444 | 909006 | RP5-1077H22.2 | Homo sapiens mRNA; cDNA DKFZp434O1214 (from clone DKFZp434O1214). (from mRNA AL137279) |
| chr6 | 958323 | 962272 | RP5-1077H22.1 | Homo sapiens cDNA clone IMAGE:8992030. (from mRNA BC126314) |
| chr6 | 1026493 | 1027225 | RP5-856G1.1 | RP5-856G1.1 (from geneSymbol) |
| chr6 | 1079928 | 1104946 | RP5-856G1.2 | Homo sapiens cDNA FLJ34594 fis, clone KIDNE2009109. (from mRNA AK091913) |
| chr6 | 1312472 | 1314187 | FOXQ1 | Homo sapiens forkhead box Q1 (FOXQ1), mRNA. (from RefSeq NM_033260) |
| chr6 | 1321697 | 1324022 | LINC01394 | long intergenic non-protein coding RNA 1394 (from HGNC LINC01394) |
| chr6 | 1383789 | 1385066 | RP4-668J24.2 | Homo sapiens cDNA clone IMAGE:6086903, partial cds. (from mRNA BC073826) |
| chr6 | 1389833 | 1395597 | FOXF2 | Homo sapiens forkhead box F2 (FOXF2), mRNA. (from RefSeq NM_001452) |
| chr6 | 1390313 | 1390411 | MIR6720 | Homo sapiens microRNA 6720 (MIR6720), microRNA. (from RefSeq NR_106778) |
