# Supplementary material for: A novel unbalanced translocation between the short arms of chromosomes 6 and 16 in a newborn girl: Clinical features and management
Source: Clin Case Rep. 2018 May 24;6(7):1282–6. doi: 10.1002/ccr3.1574 (PMC6028415; doi:10.1002/ccr3.1574)
Supplement: Supplementary file 2 [file CCR3-6-1282-s002.docx]

Table S2. List of genes duplicated in Chromosome 16 duplication: 16p13.3p12.3 (96 765-21 062 537) × 3 generated from USCS genome browser (Human Genome 38)

| chr16 | 84273 | 138860 | NPRL3 | As a component of the GATOR1 complex, inhibitor of the amino acid-sensing branch of the TORC1 pathway. The GATOR1 complex strongly increases GTP hydrolysis by RRAGA and RRAGB within RRAGC- containing heterodimers, thereby deactivating RRAGs, releasing mTORC1 from lysosomal surface and inhibiting mTORC1 signaling. (from UniProt Q12980) |
| --- | --- | --- | --- | --- |
| chr16 | 125736 | 127219 | Z69666.2 | Z69666.2 (from geneSymbol) |
| chr16 | 152686 | 154503 | HBZ | Homo sapiens hemoglobin subunit zeta (HBZ), mRNA. (from RefSeq NM_005332) |
| chr16 | 165977 | 166768 | HBM | Homo sapiens hemoglobin subunit mu (HBM), mRNA. (from RefSeq NM_001003938) |
| chr16 | 172846 | 173710 | HBA2 | Homo sapiens hemoglobin subunit alpha 2 (HBA2), mRNA. (from RefSeq NM_000517) |
| chr16 | 176679 | 177522 | HBA1 | Homo sapiens hemoglobin subunit alpha 1 (HBA1), mRNA. (from RefSeq NM_000558) |
| chr16 | 178553 | 178656 | Y_RNA | Y_RNA (from geneSymbol) |
| chr16 | 180452 | 181181 | HBQ1 | Homo sapiens hemoglobin subunit theta 1 (HBQ1), mRNA. (from RefSeq NM_005331) |
| chr16 | 185747 | 186294 | LA16c-OS12.2 | LA16c-OS12.2 (from geneSymbol) |
| chr16 | 188968 | 229450 | LUC7L | Homo sapiens LUC7-like (LUC7L), transcript variant 2, mRNA. (from RefSeq NM_201412) |
| chr16 | 234545 | 266125 | FAM234A | Homo sapiens family with sequence similarity 234 member A (FAM234A), transcript variant 1, mRNA. (from RefSeq NM_032039) |
| chr16 | 249546 | 269943 | AC004754.3 | Homo sapiens cDNA FLJ60496 complete cds. (from mRNA AK294581) |
| chr16 | 268300 | 275929 | RGS11 | Homo sapiens regulator of G-protein signaling 11 (RGS11), transcript variant 1, mRNA. (from RefSeq NM_183337) |
| chr16 | 280605 | 283010 | ARHGDIG | Homo sapiens Rho GDP dissociation inhibitor (GDI) gamma (ARHGDIG), mRNA. (from RefSeq NM_001176) |
| chr16 | 283151 | 287215 | PDIA2 | Homo sapiens protein disulfide isomerase family A member 2 (PDIA2), mRNA. (from RefSeq NM_006849) |
| chr16 | 287439 | 352659 | AXIN1 | Homo sapiens axin 1 (AXIN1), transcript variant 1, mRNA. (from RefSeq NM_003502) |
| chr16 | 367383 | 370527 | MRPL28 | Homo sapiens mitochondrial ribosomal protein L28 (MRPL28), mRNA. (from RefSeq NM_006428) |
| chr16 | 370772 | 381982 | TMEM8A | Homo sapiens transmembrane protein 8A (TMEM8A), mRNA. (from RefSeq NM_021259) |
| chr16 | 397208 | 400759 | NME4 | Homo sapiens NME/NM23 nucleoside diphosphate kinase 4 (NME4), transcript variant 1, mRNA. (from RefSeq NM_005009) |
| chr16 | 401825 | 412487 | DECR2 | Homo sapiens 2,4-dienoyl-CoA reductase 2, peroxisomal (DECR2), mRNA. (from RefSeq NM_020664) |
| chr16 | 424242 | 424543 | LA16c-359F1.1 | LA16c-359F1.1 (from geneSymbol) |
| chr16 | 425618 | 523011 | RAB11FIP3 | Homo sapiens RAB11 family interacting protein 3 (class II) (RAB11FIP3), transcript variant 1, mRNA. (from RefSeq NM_014700) |
| chr16 | 439296 | 439397 | Y_RNA | Sequence 537083 from Patent EP1572962. (from mRNA JD556059) |
| chr16 | 525154 | 527407 | LINC00235 | long intergenic non-protein coding RNA 235 (from HGNC LINC00235) |
| chr16 | 527855 | 554636 | CAPN15 | Homo sapiens calpain 15 (CAPN15), mRNA. (from RefSeq NM_005632) |
| chr16 | 535315 | 535368 | MIR5587 | Homo sapiens microRNA 5587 (MIR5587), microRNA. (from RefSeq NR_049852) |
| chr16 | 543276 | 543366 | MIR3176 | Homo sapiens microRNA 3176 (MIR3176), microRNA. (from RefSeq NR_036137) |
| chr16 | 547184 | 553847 | LA16c-366D1.3 | LA16c-366D1.3 (from geneSymbol) |
| chr16 | 560421 | 565528 | PRR35 | Homo sapiens proline rich 35 (PRR35), mRNA. (from RefSeq NM_145270) |
| chr16 | 566995 | 569487 | NHLRC4 | Homo sapiens NHL repeat containing 4 (NHLRC4), transcript variant 2, mRNA. (from RefSeq NM_176677) |
| chr16 | 568836 | 575903 | LA16c-407A10.3 | Homo sapiens cDNA, FLJ95129, highly similar to Homo sapiens phosphatidylinositol glycan, class Q (PIGQ), transcript variant 2, mRNA. (from mRNA AK314361) |
| chr16 | 570017 | 584109 | PIGQ | Homo sapiens phosphatidylinositol glycan anchor biosynthesis class Q (PIGQ), transcript variant 1, mRNA. (from RefSeq NM_148920) |
| chr16 | 589356 | 629271 | RAB40C | Homo sapiens RAB40C, member RAS oncogene family (RAB40C), transcript variant 1, mRNA. (from RefSeq NM_001172663) |
| chr16 | 630931 | 634116 | WFIKKN1 | Homo sapiens WAP, follistatin/kazal, immunoglobulin, kunitz and netrin domain containing 1 (WFIKKN1), mRNA. (from RefSeq NM_053284) |
| chr16 | 634429 | 636302 | C16orf13 | Homo sapiens chromosome 16 open reading frame 13 (C16orf13), transcript variant 1, mRNA. (from RefSeq NM_032366) |
| chr16 | 641812 | 648474 | FAM195A | Homo sapiens family with sequence similarity 195 member A (FAM195A), mRNA. (from RefSeq NM_138418) |
| chr16 | 648472 | 649200 | AL022341.3 | AL022341.3 (from geneSymbol) |
| chr16 | 649416 | 667830 | WDR90 | Homo sapiens WD repeat domain 90 (WDR90), mRNA. (from RefSeq NM_145294) |
| chr16 | 654610 | 656194 | LA16c-349E10.1 | LA16c-349E10.1 (from geneSymbol) |
| chr16 | 668085 | 674174 | RHOT2 | Homo sapiens ras homolog family member T2 (RHOT2), mRNA. (from RefSeq NM_138769) |
| chr16 | 675665 | 678268 | RHBDL1 | Homo sapiens rhomboid, veinlet-like 1 (Drosophila) (RHBDL1), transcript variant 1, mRNA. (from RefSeq NM_001278720) |
| chr16 | 678503 | 679777 | LA16c-313D11.9 | LA16c-313D11.9 (from geneSymbol) |
| chr16 | 678644 | 679061 | LA16c-313D11.13 | Homo sapiens cDNA clone IMAGE:40134351, with apparent retained intron. (from mRNA BC127824) |
| chr16 | 680275 | 682799 | STUB1 | Homo sapiens STIP1 homology and U-box containing protein 1, E3 ubiquitin protein ligase (STUB1), transcript variant 1, mRNA. (from RefSeq NM_005861) |
| chr16 | 681670 | 684439 | JMJD8 | Homo sapiens jumonji domain containing 8 (JMJD8), mRNA. (from RefSeq NM_001005920) |
| chr16 | 684621 | 690400 | WDR24 | Homo sapiens WD repeat domain 24 (WDR24), mRNA. (from RefSeq NM_032259) |
| chr16 | 689493 | 692554 | LA16c-313D11.12 | LA16c-313D11.12 (from geneSymbol) |
| chr16 | 692499 | 705829 | FBXL16 | Homo sapiens F-box and leucine-rich repeat protein 16 (FBXL16), mRNA. (from RefSeq NM_153350) |
| chr16 | 707649 | 709067 | LA16c-380A1.2 | Homo sapiens cDNA FLJ52617 complete cds. (from mRNA AK304832) |
| chr16 | 710745 | 711277 | LA16c-380A1.1 | LA16c-380A1.1 (from geneSymbol) |
| chr16 | 715114 | 719655 | METRN | Homo sapiens meteorin, glial cell differentiation regulator (METRN), mRNA. (from RefSeq NM_024042) |
| chr16 | 720974 | 722601 | FAM173A | Homo sapiens family with sequence similarity 173 member A (FAM173A), transcript variant 1, mRNA. (from RefSeq NM_023933) |
| chr16 | 722581 | 726473 | CCDC78 | Homo sapiens coiled-coil domain containing 78 (CCDC78), mRNA. (from RefSeq NM_001031737) |
| chr16 | 727270 | 729715 | HAGHL | Homo sapiens hydroxyacylglutathione hydrolase-like (HAGHL), transcript variant 2, mRNA. (from RefSeq NM_032304) |
| chr16 | 729752 | 741002 | NARFL | Homo sapiens nuclear prelamin A recognition factor-like (NARFL), transcript variant 1, mRNA. (from RefSeq NM_022493) |
| chr16 | 760761 | 768862 | MSLN | Homo sapiens mesothelin (MSLN), transcript variant 3, mRNA. (from RefSeq NM_001177355) |
| chr16 | 769118 | 769443 | LA16c-335H7.2 | Sequence 465785 from Patent EP1572962. (from mRNA JD484761) |
| chr16 | 769427 | 782054 | MSLNL | The sequence shown here is derived from an Ensembl automatic analysis pipeline and should be considered as preliminary data. (from UniProt H0YG18) |
| chr16 | 770182 | 770277 | MIR662 | Homo sapiens microRNA 662 (MIR662), microRNA. (from RefSeq NR_030384) |
| chr16 | 784973 | 788383 | RPUSD1 | Homo sapiens RNA pseudouridylate synthase domain containing 1 (RPUSD1), mRNA. (from RefSeq NM_058192) |
| chr16 | 788621 | 798074 | CHTF18 | Homo sapiens chromosome transmission fidelity factor 18 (CHTF18), mRNA. (from RefSeq NM_022092) |
| chr16 | 798040 | 800733 | GNG13 | Homo sapiens guanine nucleotide binding protein (G protein), gamma 13 (GNG13), mRNA. (from RefSeq NM_016541) |
| chr16 | 805442 | 813861 | PRR25 | Homo sapiens proline rich 25 (PRR25), mRNA. (from RefSeq NM_001013638) |
| chr16 | 848524 | 849065 | LA16c-360A4.1 | LA16c-360A4.1 (from geneSymbol) |
| chr16 | 853633 | 970999 | LMF1 | Homo sapiens lipase maturation factor 1 (LMF1), transcript variant 1, mRNA. (from RefSeq NM_022773) |
| chr16 | 883779 | 885090 | LA16c-306A4.1 | LA16c-306A4.1 (from geneSymbol) |
| chr16 | 898966 | 905224 | LA16c-306A4.2 | Homo sapiens cDNA FLJ43617 fis, clone SPLEN2016863. (from mRNA AK125605) |
| chr16 | 921072 | 934495 | LMF1-AS1 | Homo sapiens LMF1 antisense RNA 1 (LMF1-AS1), transcript variant 1, long non-coding RNA. (from RefSeq NR_110945) |
| chr16 | 968374 | 969012 | RP11-161M6.6 | RP11-161M6.6 (from geneSymbol) |
| chr16 | 975760 | 981590 | RP11-161M6.2 | Homo sapiens cDNA clone IMAGE:5264670. (from mRNA BC036550) |
| chr16 | 981807 | 986979 | SOX8 | Homo sapiens SRY-box 8 (SOX8), mRNA. (from RefSeq NM_014587) |
| chr16 | 991150 | 1000926 | RP11-161M6.3 | RP11-161M6.3 (from geneSymbol) |
| chr16 | 1064092 | 1078673 | SSTR5-AS1 | Homo sapiens SSTR5 antisense RNA 1 (SSTR5-AS1), long non-coding RNA. (from RefSeq NR_027242) |
| chr16 | 1065239 | 1066502 | RP11-161M6.5 | RP11-161M6.5 (from geneSymbol) |
| chr16 | 1078780 | 1080142 | SSTR5 | Homo sapiens somatostatin receptor 5 (SSTR5), transcript variant 2, mRNA. (from RefSeq NM_001172560) |
| chr16 | 1090004 | 1096244 | C1QTNF8 | Homo sapiens C1q and tumor necrosis factor related protein 8 (C1QTNF8), mRNA. (from RefSeq NM_207419) |
| chr16 | 1111626 | 1113399 | LA16c-381G6.1 | Sequence 248449 from Patent EP1572962. (from mRNA JD267425) |
| chr16 | 1148223 | 1148754 | RP11-616M22.11 | RP11-616M22.11 (from geneSymbol) |
| chr16 | 1153240 | 1221768 | CACNA1H | Homo sapiens calcium voltage-gated channel subunit alpha1 H (CACNA1H), transcript variant 1, mRNA. (from RefSeq NM_021098) |
| chr16 | 1156975 | 1157974 | RP11-616M22.1 | RP11-616M22.1 (from geneSymbol) |
| chr16 | 1159547 | 1160176 | RP11-616M22.2 | RP11-616M22.2 (from geneSymbol) |
| chr16 | 1206559 | 1207124 | RP11-616M22.3 | RP11-616M22.3 (from geneSymbol) |
| chr16 | 1221650 | 1225257 | TPSG1 | Homo sapiens tryptase gamma 1 (TPSG1), mRNA. (from RefSeq NM_012467) |
| chr16 | 1223638 | 1224143 | RP11-616M22.12 | RP11-616M22.12 (from geneSymbol) |
| chr16 | 1228336 | 1230162 | TPSB2 | Belongs to the peptidase S1 family. (from UniProt A0A087WUI4) |
| chr16 | 1240695 | 1242554 | TPSAB1 | Homo sapiens tryptase alpha/beta 1 (TPSAB1), mRNA. (from RefSeq NM_003294) |
| chr16 | 1256132 | 1258998 | TPSD1 | Homo sapiens tryptase delta 1 (TPSD1), mRNA. (from RefSeq NM_012217) |
| chr16 | 1257338 | 1258074 | RP11-616M22.5 | RP11-616M22.5 (from geneSymbol) |
| chr16 | 1294550 | 1299166 | RP11-616M22.7 | RP11-616M22.7 (from geneSymbol) |
| chr16 | 1305546 | 1309413 | LA16c-358B7.4 | Sequence 539301 from Patent EP1572962. (from mRNA JD558277) |
| chr16 | 1308879 | 1327018 | UBE2I | Homo sapiens ubiquitin conjugating enzyme E2I (UBE2I), transcript variant 3, mRNA. (from RefSeq NM_194260) |
| chr16 | 1317890 | 1322845 | LA16c-358B7.3 | LA16c-358B7.3 (from geneSymbol) |
| chr16 | 1334503 | 1349438 | BAIAP3 | Homo sapiens BAI1 associated protein 3 (BAIAP3), transcript variant 1, mRNA. (from RefSeq NM_003933) |
| chr16 | 1349239 | 1351911 | TSR3 | Homo sapiens TSR3, 20S rRNA accumulation, homolog (S. cerevisiae) (TSR3), mRNA. (from RefSeq NM_001001410) |
| chr16 | 1351922 | 1364113 | GNPTG | Homo sapiens N-acetylglucosamine-1-phosphate transferase, gamma subunit (GNPTG), mRNA. (from RefSeq NM_032520) |
| chr16 | 1358899 | 1361405 | LA16c-316G12.2 | LA16c-316G12.2 (from geneSymbol) |
| chr16 | 1365011 | 1414691 | UNKL | Contains RING-type zinc finger. (from UniProt E9PDK2) |
| chr16 | 1379288 | 1379423 | AL031721.1 | AL031721.1 (from geneSymbol) |
| chr16 | 1408833 | 1412248 | LA16c-312E8.2 | LA16c-312E8.2 (from geneSymbol) |
| chr16 | 1419743 | 1420800 | C16orf91 | Homo sapiens chromosome 16 open reading frame 91 (C16orf91), mRNA. (from RefSeq NM_001272051) |
| chr16 | 1434387 | 1444489 | CCDC154 | Homo sapiens coiled-coil domain containing 154 (CCDC154), mRNA. (from RefSeq NM_001143980) |
| chr16 | 1437153 | 1439315 | LA16c-312E8.4 | Homo sapiens cDNA clone IMAGE:4111037, partial cds. (from mRNA BC011663) |
| chr16 | 1444934 | 1475580 | CLCN7 | Homo sapiens chloride voltage-gated channel 7 (CLCN7), transcript variant 1, mRNA. (from RefSeq NM_001287) |
| chr16 | 1445342 | 1446519 | LA16c-390E6.5 | LA16c-390E6.5 (from geneSymbol) |
| chr16 | 1451759 | 1452653 | LA16c-390E6.4 | LA16c-390E6.4 (from geneSymbol) |
| chr16 | 1467672 | 1472684 | LA16c-390E6.3 | LA16c-390E6.3 (from geneSymbol) |
| chr16 | 1485938 | 1488467 | PTX4 | Homo sapiens pentraxin 4 (PTX4), mRNA. (from RefSeq NM_001013658) |
| chr16 | 1493362 | 1510457 | TELO2 | Homo sapiens telomere maintenance 2 (TELO2), mRNA. (from RefSeq NM_016111) |
| chr16 | 1510426 | 1612110 | IFT140 | Homo sapiens intraflagellar transport 140 (IFT140), mRNA. (from RefSeq NM_014714) |
| chr16 | 1512978 | 1514675 | LA16c-385E7.1 | LA16c-385E7.1 (from geneSymbol) |
| chr16 | 1528687 | 1555580 | TMEM204 | Homo sapiens transmembrane protein 204 (TMEM204), transcript variant 2, mRNA. (from RefSeq NM_001256541) |
| chr16 | 1530714 | 1533301 | LA16c-380F5.1 | Homo sapiens cDNA clone IMAGE:40068941. (from mRNA BC117675) |
| chr16 | 1553654 | 1554130 | LA16c-380F5.3 | Homo sapiens cDNA clone IMAGE:40010430. (from mRNA BC112436) |
| chr16 | 1576715 | 1578371 | LA16c-313F4.1 | Sequence 7467 from Patent EP1572962. (from mRNA JD026443) |
| chr16 | 1579241 | 1580308 | LA16c-425C2.1 | LA16c-425C2.1 (from geneSymbol) |
| chr16 | 1580526 | 1610328 | LA16c-395F10.2 | LA16c-395F10.2 (from geneSymbol) |
| chr16 | 1612324 | 1677908 | CRAMP1 | Nucleus (from UniProt Q96RY5) |
| chr16 | 1625627 | 1626160 | LA16c-395F10.1 | LA16c-395F10.1 (from geneSymbol) |
| chr16 | 1632258 | 1686715 | LA16c-431H6.6 | The sequence shown here is derived from an Ensembl automatic analysis pipeline and should be considered as preliminary data. (from UniProt J3QT63) |
| chr16 | 1678255 | 1702086 | HN1L | Homo sapiens hematological and neurological expressed 1-like (HN1L), mRNA. (from RefSeq NM_144570) |
| chr16 | 1688354 | 1690536 | LA16c-431H6.7 | Sequence 247545 from Patent EP1572962. (from mRNA JD266521) |
| chr16 | 1706182 | 1770317 | MAPK8IP3 | Homo sapiens mitogen-activated protein kinase 8 interacting protein 3 (MAPK8IP3), transcript variant 1, mRNA. (from RefSeq NM_015133) |
| chr16 | 1707251 | 1707973 | LA16c-329F2.2 | LA16c-329F2.2 (from geneSymbol) |
| chr16 | 1713526 | 1714208 | LA16c-329F2.1 | LA16c-329F2.1 (from geneSymbol) |
| chr16 | 1734984 | 1735066 | MIR3177 | Homo sapiens microRNA 3177 (MIR3177), microRNA. (from RefSeq NR_036138) |
| chr16 | 1751558 | 1752262 | LA16c-361A3.3 | LA16c-361A3.3 (from geneSymbol) |
| chr16 | 1770285 | 1771730 | NME3 | Homo sapiens NME/NM23 nucleoside diphosphate kinase 3 (NME3), mRNA. (from RefSeq NM_002513) |
| chr16 | 1771889 | 1773155 | MRPS34 | Homo sapiens mitochondrial ribosomal protein S34 (MRPS34), transcript variant 2, mRNA. (from RefSeq NM_023936) |
| chr16 | 1773206 | 1776311 | EME2 | Homo sapiens essential meiotic structure-specific endonuclease subunit 2 (EME2), mRNA. (from RefSeq NM_001257370) |
| chr16 | 1776711 | 1782820 | SPSB3 | Homo sapiens splA/ryanodine receptor domain and SOCS box containing 3 (SPSB3), mRNA. (from RefSeq NM_080861) |
| chr16 | 1782900 | 1789191 | NUBP2 | Homo sapiens nucleotide binding protein 2 (NUBP2), transcript variant 1, mRNA. (from RefSeq NM_012225) |
| chr16 | 1790412 | 1793763 | IGFALS | Homo sapiens insulin like growth factor binding protein acid labile subunit (IGFALS), transcript variant 2, mRNA. (from RefSeq NM_004970) |
| chr16 | 1807632 | 1827194 | HAGH | Homo sapiens hydroxyacylglutathione hydrolase (HAGH), transcript variant 1, mRNA. (from RefSeq NM_005326) |
| chr16 | 1826940 | 1828914 | FAHD1 | Homo sapiens fumarylacetoacetate hydrolase domain containing 1 (FAHD1), transcript variant 2, mRNA. (from RefSeq NM_031208) |
| chr16 | 1833987 | 1872178 | MEIOB | Homo sapiens meiosis specific with OB domains (MEIOB), transcript variant 2, mRNA. (from RefSeq NM_152764) |
| chr16 | 1841019 | 1843547 | LA16c-429E7.1 | LA16c-429E7.1 (from geneSymbol) |
| chr16 | 1878284 | 1884231 | LINC00254 | Homo sapiens long intergenic non-protein coding RNA 254 (LINC00254), long non-coding RNA. (from RefSeq NR_033914) |
| chr16 | 1889113 | 1890434 | XX-DJ76P10__A.2 | XX-DJ76P10__A.2 (from geneSymbol) |
| chr16 | 1911462 | 1918440 | HS3ST6 | Homo sapiens heparan sulfate-glucosamine 3-sulfotransferase 6 (HS3ST6), mRNA. (from RefSeq NM_001009606) |
| chr16 | 1938209 | 1943326 | MSRB1 | Homo sapiens methionine sulfoxide reductase B1 (MSRB1), mRNA. (from RefSeq NM_016332) |
| chr16 | 1943973 | 1954726 | RPL3L | Homo sapiens ribosomal protein L3 like (RPL3L), mRNA. (from RefSeq NM_005061) |
| chr16 | 1959507 | 1961975 | NDUFB10 | Homo sapiens NADH:ubiquinone oxidoreductase subunit B10 (NDUFB10), mRNA. (from RefSeq NM_004548) |
| chr16 | 1962051 | 1964860 | RPS2 | Homo sapiens ribosomal protein S2 (RPS2), mRNA. (from RefSeq NM_002952) |
| chr16 | 1962333 | 1962466 | SNORA10 | Homo sapiens small nucleolar RNA, H/ACA box 10 (SNORA10), small nucleolar RNA. (from RefSeq NR_002327) |
| chr16 | 1962972 | 1963106 | SNORA64 | Homo sapiens small nucleolar RNA, H/ACA box 64 (SNORA64), small nucleolar RNA. (from RefSeq NR_002326) |
| chr16 | 1964958 | 1965509 | SNHG9 | small nucleolar RNA host gene 9 (from HGNC SNHG9) |
| chr16 | 1965183 | 1965310 | SNORA78 | Homo sapiens small nucleolar RNA, H/ACA box 78 (SNORA78), small nucleolar RNA. (from RefSeq NR_003020) |
| chr16 | 1966873 | 1968975 | RNF151 | Homo sapiens ring finger protein 151 (RNF151), mRNA. (from RefSeq NM_174903) |
| chr16 | 1971654 | 1971896 | AC005363.11 | AC005363.11 (from geneSymbol) |
| chr16 | 1972036 | 1982933 | TBL3 | Homo sapiens transducin (beta)-like 3 (TBL3), mRNA. (from RefSeq NM_006453) |
| chr16 | 1979039 | 1981183 | NOXO1 | Homo sapiens NADPH oxidase organizer 1 (NOXO1), transcript variant c, mRNA. (from RefSeq NM_172168) |
| chr16 | 1984212 | 1987749 | GFER | Homo sapiens growth factor, augmenter of liver regeneration (GFER), mRNA. (from RefSeq NM_005262) |
| chr16 | 1984876 | 1986853 | AC005606.14 | AC005606.14 (from geneSymbol) |
| chr16 | 1989944 | 1994275 | SYNGR3 | Homo sapiens synaptogyrin 3 (SYNGR3), mRNA. (from RefSeq NM_004209) |
| chr16 | 1997653 | 1998374 | AC005606.15 | AC005606.15 (from geneSymbol) |
| chr16 | 1997654 | 2009821 | ZNF598 | Homo sapiens zinc finger protein 598 (ZNF598), mRNA. (from RefSeq NM_178167) |
| chr16 | 2013180 | 2013476 | RN7SL219P | RNA, 7SL, cytoplasmic 219, pseudogene (from HGNC RN7SL219P) |
| chr16 | 2019519 | 2020754 | NPW | Homo sapiens neuropeptide W (NPW), mRNA. (from RefSeq NM_001099456) |
| chr16 | 2026867 | 2039026 | SLC9A3R2 | Homo sapiens SLC9A3 regulator 2 (SLC9A3R2), transcript variant 1, mRNA. (from RefSeq NM_001130012) |
| chr16 | 2039814 | 2047866 | NTHL1 | Homo sapiens nth-like DNA glycosylase 1 (NTHL1), transcript variant 1, mRNA. (from RefSeq NM_002528) |
| chr16 | 2047464 | 2088712 | TSC2 | Homo sapiens tuberous sclerosis 2 (TSC2), transcript variant 1, mRNA. (from RefSeq NM_000548) |
| chr16 | 2088709 | 2135898 | PKD1 | Homo sapiens polycystin 1, transient receptor potential channel interacting (PKD1), transcript variant 1, mRNA. (from RefSeq NM_001009944) |
| chr16 | 2090194 | 2090284 | MIR1225 | Homo sapiens microRNA 1225 (MIR1225), microRNA. (from RefSeq NR_030646) |
| chr16 | 2091538 | 2095433 | RP11-304L19.1 | RP11-304L19.1 (from geneSymbol) |
| chr16 | 2094829 | 2097026 | RP11-304L19.3 | RP11-304L19.3 (from geneSymbol) |
| chr16 | 2102256 | 2102316 | AC009065.3 | AC009065.3 (from geneSymbol) |
| chr16 | 2106668 | 2106753 | MIR6511B1 | Homo sapiens microRNA 6511b-1 (MIR6511B1), microRNA. (from RefSeq NR_106775) |
| chr16 | 2112334 | 2113342 | RP11-304L19.4 | RP11-304L19.4 (from geneSymbol) |
| chr16 | 2119206 | 2120248 | RP11-304L19.2 | RP11-304L19.2 (from geneSymbol) |
| chr16 | 2131850 | 2131950 | Y_RNA | Y_RNA (from geneSymbol) |
| chr16 | 2133118 | 2133204 | MIR4516 | Homo sapiens microRNA 4516 (MIR4516), microRNA. (from RefSeq NR_039741) |
| chr16 | 2135976 | 2136129 | MIR3180-5 | Homo sapiens microRNA 3180-5 (MIR3180-5), microRNA. (from RefSeq NR_037467) |
| chr16 | 2148623 | 2154165 | RAB26 | Homo sapiens RAB26, member RAS oncogene family (RAB26), transcript variant 1, mRNA. (from RefSeq NM_014353) |
| chr16 | 2154796 | 2155358 | SNHG19 | Homo sapiens small nucleolar RNA host gene 19 (SNHG19), long non-coding RNA. (from RefSeq NR_132114) |
| chr16 | 2155022 | 2155105 | SNORD60 | Homo sapiens small nucleolar RNA, C/D box 60 (SNORD60), small nucleolar RNA. (from RefSeq NR_002736) |
| chr16 | 2155024 | 2155104 | snoR1 | H.sapiens mRNA for U60 small nuclear RNA. (from mRNA X96660) |
| chr16 | 2155764 | 2178129 | TRAF7 | Homo sapiens TNF receptor associated factor 7 (TRAF7), mRNA. (from RefSeq NM_032271) |
| chr16 | 2177179 | 2196525 | CASKIN1 | Homo sapiens CASK interacting protein 1 (CASKIN1), mRNA. (from RefSeq NM_020764) |
| chr16 | 2205029 | 2205136 | AC009065.2 | AC009065.2 (from geneSymbol) |
| chr16 | 2205176 | 2209396 | MLST8 | Homo sapiens MTOR associated protein, LST8 homolog (MLST8), transcript variant 2, mRNA. (from RefSeq NM_001199173) |
| chr16 | 2209252 | 2211950 | BRICD5 | Homo sapiens BRICHOS domain containing 5 (BRICD5), mRNA. (from RefSeq NM_182563) |
| chr16 | 2211996 | 2212863 | RP11-304L19.8 | RP11-304L19.8 (from geneSymbol) |
| chr16 | 2211996 | 2214807 | PGP | Homo sapiens phosphoglycolate phosphatase (PGP), mRNA. (from RefSeq NM_001042371) |
| chr16 | 2223565 | 2235742 | E4F1 | Homo sapiens E4F transcription factor 1 (E4F1), transcript variant 1, mRNA. (from RefSeq NM_004424) |
| chr16 | 2235688 | 2236913 | RP11-304L19.12 | Homo sapiens E4F transcription factor 1, mRNA (cDNA clone IMAGE:3353716), partial cds. (from mRNA BC001713) |
| chr16 | 2235815 | 2238711 | DNASE1L2 | Divalent cation-dependent acid DNA endonuclease involved in the breakdown of the nucleus during corneocyte formation of epidermal keratinocytes. May play an immune role by eliminating harmful DNA released into the extracellular environment by damaged epidermal cells. (from UniProt Q92874) |
| chr16 | 2239394 | 2251614 | ECI1 | Homo sapiens enoyl-CoA delta isomerase 1 (ECI1), transcript variant 1, mRNA. (from RefSeq NM_001919) |
| chr16 | 2240486 | 2241818 | RP11-304L19.11 | RP11-304L19.11 (from geneSymbol) |
| chr16 | 2253115 | 2268412 | RNPS1 | Homo sapiens RNA binding protein S1, serine-rich domain (RNPS1), transcript variant 2, mRNA. (from RefSeq NM_080594) |
| chr16 | 2253736 | 2262342 | AC009065.4 | The sequence shown here is derived from an Ensembl automatic analysis pipeline and should be considered as preliminary data. (from UniProt A0A096LP58) |
| chr16 | 2267786 | 2267880 | AC009065.1 | DETECTING NUCLEIC ACIDS. (from mRNA FW312332) |
| chr16 | 2268154 | 2273418 | RP11-304L19.13 | RP11-304L19.13 (from geneSymbol) |
| chr16 | 2270712 | 2270772 | MIR3677 | Homo sapiens microRNA 3677 (MIR3677), microRNA. (from RefSeq NR_037448) |
| chr16 | 2271736 | 2271846 | MIR940 | Homo sapiens microRNA 940 (MIR940), microRNA. (from RefSeq NR_030636) |
| chr16 | 2274619 | 2274691 | MIR4717 | Homo sapiens microRNA 4717 (MIR4717), microRNA. (from RefSeq NR_039868) |
| chr16 | 2275880 | 2340735 | ABCA3 | Homo sapiens ATP binding cassette subfamily A member 3 (ABCA3), mRNA. (from RefSeq NM_001089) |
| chr16 | 2429393 | 2458854 | CCNF | Homo sapiens cyclin F (CCNF), mRNA. (from RefSeq NM_001761) |
| chr16 | 2452580 | 2452977 | RP11-715J22.3 | Homo sapiens cDNA clone IMAGE:5106201, partial cds. (from mRNA BC035887) |
| chr16 | 2456251 | 2459966 | RP11-715J22.4 | RP11-715J22.4 (from geneSymbol) |
| chr16 | 2460079 | 2464963 | C16orf59 | Homo sapiens chromosome 16 open reading frame 59 (C16orf59), mRNA. (from RefSeq NM_025108) |
| chr16 | 2463966 | 2464038 | MIR6768 | Homo sapiens microRNA 6768 (MIR6768), microRNA. (from RefSeq NR_106826) |
| chr16 | 2464949 | 2468213 | RP11-715J22.2 | Homo sapiens mRNA; cDNA DKFZp434L055 (from clone DKFZp434L055). (from mRNA AL834536) |
| chr16 | 2471498 | 2474145 | NTN3 | Homo sapiens netrin 3 (NTN3), mRNA. (from RefSeq NM_006181) |
| chr16 | 2475145 | 2505734 | TBC1D24 | Homo sapiens TBC1 domain family member 24 (TBC1D24), transcript variant 2, mRNA. (from RefSeq NM_020705) |
| chr16 | 2476557 | 2482173 | RP11-715J22.6 | Homo sapiens full length insert cDNA clone YB25F11. (from mRNA AF147335) |
| chr16 | 2496031 | 2520218 | RP11-20I23.1 | The sequence shown here is derived from an Ensembl automatic analysis pipeline and should be considered as preliminary data. (from UniProt H3BQ06) |
| chr16 | 2513869 | 2520218 | ATP6V0C | Homo sapiens ATPase H+ transporting V0 subunit c (ATP6V0C), transcript variant 1, mRNA. (from RefSeq NM_001694) |
| chr16 | 2513964 | 2527955 | RP11-20I23.3 | The sequence shown here is derived from an Ensembl automatic analysis pipeline and should be considered as preliminary data. (from UniProt H3BQ15) |
| chr16 | 2516657 | 2517999 | RP11-20I23.2 | Sequence 237028 from Patent EP1572962. (from mRNA JD256004) |
| chr16 | 2520364 | 2529698 | AMDHD2 | Hydrolyzes the N-glycolyl group from N- glycolylglucosamine 6-phosphate (GlcNGc-6-P) in the N- glycolylneuraminic acid (Neu5Gc) degradation pathway. Although human is not able to catalyze formation of Neu5Gc due to the inactive CMAHP enzyme, Neu5Gc is present in food and must be degraded. (from UniProt Q9Y303) |
| chr16 | 2530034 | 2531408 | CEMP1 | Homo sapiens cementum protein 1 (CEMP1), mRNA. (from RefSeq NM_001048212) |
| chr16 | 2531921 | 2532005 | MIR3178 | Homo sapiens microRNA 3178 (MIR3178), microRNA. (from RefSeq NR_036139) |
| chr16 | 2537963 | 2603188 | PDPK1 | Homo sapiens 3-phosphoinositide dependent protein kinase 1 (PDPK1), transcript variant 1, mRNA. (from RefSeq NM_002613) |
| chr16 | 2554059 | 2556060 | RP11-20I23.13 | Homo sapiens cDNA FLJ38998 fis, clone NT2RI2021463. (from mRNA AK096317) |
| chr16 | 2554974 | 2556105 | RP11-20I23.11 | RP11-20I23.11 (from geneSymbol) |
| chr16 | 2561470 | 2565096 | RP11-20I23.8 | Homo sapiens cDNA FLJ12913 fis, clone NT2RP2004490. (from mRNA AK022975) |
| chr16 | 2569042 | 2571936 | RP11-20I23.6 | RP11-20I23.6 (from geneSymbol) |
| chr16 | 2571569 | 2572353 | RP11-20I23.7 | RP11-20I23.7 (from geneSymbol) |
| chr16 | 2575627 | 2577373 | RP11-20I23.5 | RP11-20I23.5 (from geneSymbol) |
| chr16 | 2578394 | 2579963 | RP11-20I23.10 | Homo sapiens cDNA clone IMAGE:6278842, partial cds. (from mRNA BC051280) |
| chr16 | 2592513 | 2594563 | CTD-3126B10.2 | Homo sapiens cDNA FLJ36368 fis, clone THYMU2007969. (from mRNA AK093687) |
| chr16 | 2597880 | 2599718 | CTD-3126B10.1 | Homo sapiens piRNA piR-33835, complete sequence. (from mRNA DQ593723) |
| chr16 | 2620221 | 2621794 | CTD-3126B10.5 | CTD-3126B10.5 (from geneSymbol) |
| chr16 | 2644083 | 2645214 | CTD-3126B10.4 | CTD-3126B10.4 (from geneSymbol) |
| chr16 | 2660348 | 2673374 | CTD-2270P14.1 | Homo sapiens cDNA clone IMAGE:5105626, partial cds. (from mRNA BC063714) |
| chr16 | 2682474 | 2709030 | KCTD5 | Homo sapiens potassium channel tetramerization domain containing 5 (KCTD5), mRNA. (from RefSeq NM_018992) |
| chr16 | 2683105 | 2684764 | CTD-2270P14.2 | Sequence 121155 from Patent EP1572962. (from mRNA JD140131) |
| chr16 | 2712417 | 2720215 | PRSS27 | Homo sapiens protease, serine 27 (PRSS27), transcript variant 1, mRNA. (from RefSeq NM_031948) |
| chr16 | 2713018 | 2713100 | AC092117.1 | AC092117.1 (from geneSymbol) |
| chr16 | 2737102 | 2752600 | SRRM2-AS1 | Homo sapiens SRRM2 antisense RNA 1 (SRRM2-AS1), transcript variant 2, long non-coding RNA. (from RefSeq NR_027275) |
| chr16 | 2752328 | 2771412 | SRRM2 | Homo sapiens serine/arginine repetitive matrix 2 (SRRM2), mRNA. (from RefSeq NM_016333) |
| chr16 | 2769871 | 2769949 | AC092117.2 | AC092117.2 (from geneSymbol) |
| chr16 | 2771413 | 2777297 | TCEB2 | Homo sapiens transcription elongation factor B (SIII), polypeptide 2 (18 kDa, elongin B) (TCEB2), transcript variant 1, mRNA. (from RefSeq NM_007108) |
| chr16 | 2777318 | 2780568 | CTD-2270P14.5 | Homo sapiens cDNA FLJ31889 fis, clone NT2RP7003091. (from mRNA AK056451) |
| chr16 | 2783952 | 2787948 | PRSS33 | Serine protease that has amidolytic activity, cleaving its substrates before Arg residues. (from UniProt Q8NF86) |
| chr16 | 2796407 | 2796532 | SNORA3 | SNORA3 (from geneSymbol) |
| chr16 | 2817226 | 2821719 | PRSS21 | Homo sapiens protease, serine 21 (PRSS21), transcript variant 2, mRNA. (from RefSeq NM_144956) |
| chr16 | 2830168 | 2832284 | ZG16B | Homo sapiens zymogen granule protein 16B (ZG16B), mRNA. (from RefSeq NM_145252) |
| chr16 | 2852726 | 2858170 | PRSS22 | Homo sapiens protease, serine 22 (PRSS22), mRNA. (from RefSeq NM_022119) |
| chr16 | 2857897 | 2859726 | LA16c-325D7.1 | LA16c-325D7.1 (from geneSymbol) |
| chr16 | 2866347 | 2867618 | LA16c-325D7.2 | LA16c-325D7.2 (from geneSymbol) |
| chr16 | 2883185 | 2899382 | FLYWCH2 | Homo sapiens FLYWCH family member 2 (FLYWCH2), transcript variant 1, mRNA. (from RefSeq NM_138439) |
| chr16 | 2911998 | 2951199 | FLYWCH1 | Homo sapiens FLYWCH-type zinc finger 1 (FLYWCH1), transcript variant 1, mRNA. (from RefSeq NM_032296) |
| chr16 | 2939713 | 2954276 | LA16c-321D4.2 | LA16c-321D4.2 (from geneSymbol) |
| chr16 | 2963943 | 2968380 | KREMEN2 | Homo sapiens kringle containing transmembrane protein 2 (KREMEN2), transcript variant 4, mRNA. (from RefSeq NM_172229) |
| chr16 | 2969244 | 2973489 | PAQR4 | Homo sapiens progestin and adipoQ receptor family member IV (PAQR4), transcript variant 1, mRNA. (from RefSeq NM_152341) |
| chr16 | 2972850 | 2980479 | PKMYT1 | Acts as a negative regulator of entry into mitosis (G2 to M transition) by phosphorylation of the CDK1 kinase specifically when CDK1 is complexed to cyclins. Mediates phosphorylation of CDK1 predominantly on “Thr-14”. Also involved in Golgi fragmentation. May be involved in phosphorylation of CDK1 on “Tyr-15” to a lesser degree, however tyrosine kinase activity is unclear and may be indirect. May be a downstream target of Notch signaling pathway during eye development. (from UniProt Q99640) |
| chr16 | 2981174 | 2981591 | LA16c-380H5.6 | Sequence 453008 from Patent EP1572962. (from mRNA JD471984) |
| chr16 | 2988255 | 3002016 | LA16c-380H5.3 | Homo sapiens RNA.p13-3.b RNA, partial sequence. (from mRNA HM587423) |
| chr16 | 2988960 | 2992959 | LA16c-380H5.1 | Homo sapiens cDNA FLJ40698 fis, clone THYMU2025557. (from mRNA AK098017) |
| chr16 | 3003430 | 3005101 | LA16c-380H5.2 | LA16c-380H5.2 (from geneSymbol) |
| chr16 | 3006119 | 3007388 | LA16c-380H5.5 | LA16c-380H5.5 (from geneSymbol) |
| chr16 | 3012455 | 3014505 | CLDN9 | Homo sapiens claudin 9 (CLDN9), mRNA. (from RefSeq NM_020982) |
| chr16 | 3014711 | 3020071 | CLDN6 | Plays a major role in tight junction-specific obliteration of the intercellular space (By similarity). May act as a coreceptor for HCV entry into hepatic cells. (from UniProt P56747) |
| chr16 | 3020311 | 3022383 | TNFRSF12A | Homo sapiens tumor necrosis factor receptor superfamily member 12A (TNFRSF12A), mRNA. (from RefSeq NM_016639) |
| chr16 | 3022624 | 3023961 | HCFC1R1 | Homo sapiens host cell factor C1 regulator 1 (XPO1 dependent) (HCFC1R1), transcript variant 5, mRNA. (from RefSeq NM_001288666) |
| chr16 | 3024030 | 3027754 | THOC6 | Homo sapiens THO complex 6 (THOC6), transcript variant 1, mRNA. (from RefSeq NM_024339) |
| chr16 | 3027711 | 3036926 | CCDC64B | Interacts with RAB13. (from UniProt A1A5D9) |
| chr16 | 3032480 | 3039133 | RP11-473M20.5 | Homo sapiens uncharacterized LOC100128770 (LOC100128770), long non-coding RNA. (from RefSeq NR_047572) |
| chr16 | 3046680 | 3060726 | MMP25 | Homo sapiens matrix metallopeptidase 25 (MMP25), mRNA. (from RefSeq NM_022468) |
| chr16 | 3051095 | 3056232 | MMP25-AS1 | MMP25 antisense RNA 1 (from HGNC MMP25-AS1) |
| chr16 | 3065311 | 3069551 | IL32 | Cytokine that may play a role in innate and adaptive immune responses. It induces various cytokines such as TNFA/TNF- alpha and IL8. It activates typical cytokine signal pathways of NF-kappa-B and p38 MAPK. (from UniProt P24001) |
| chr16 | 3069522 | 3069651 | RNU1-125P | RNA, U1 small nuclear 125, pseudogene (from HGNC RNU1-125P) |
| chr16 | 3076910 | 3087100 | RP11-473M20.9 | Homo sapiens cDNA clone IMAGE:5201917, partial cds. (from mRNA BC046244) |
| chr16 | 3086142 | 3086300 | RNU1-22P | RNA, U1 small nuclear 22, pseudogene (from HGNC RNU1-22P) |
| chr16 | 3088889 | 3099317 | ZSCAN10 | Homo sapiens zinc finger and SCAN domain containing 10 (ZSCAN10), transcript variant 1, mRNA. (from RefSeq NM_032805) |
| chr16 | 3106763 | 3109576 | RP11-473M20.11 | RP11-473M20.11 (from geneSymbol) |
| chr16 | 3110459 | 3134869 | ZNF213-AS1 | ZNF213 antisense RNA 1 (head to head) (from HGNC ZNF213-AS1) |
| chr16 | 3112559 | 3120517 | ZNF205 | Homo sapiens zinc finger protein 205 (ZNF205), transcript variant 1, mRNA. (from RefSeq NM_003456) |
| chr16 | 3134922 | 3142803 | ZNF213 | May be involved in transcriptional regulation. (from UniProt O14771) |
| chr16 | 3156735 | 3157483 | RP11-473M20.16 | Homo sapiens piRNA piR-35413, complete sequence. (from mRNA DQ597347) |
| chr16 | 3181232 | 3184018 | AJ003147.8 | AJ003147.8 (from geneSymbol) |
| chr16 | 3188211 | 3224779 | AJ003147.9 | AJ003147.9 (from geneSymbol) |
| chr16 | 3204246 | 3205188 | OR1F1 | Homo sapiens olfactory receptor family 1 subfamily F member 1 (OR1F1), mRNA. (from RefSeq NM_012360) |
| chr16 | 3222324 | 3235456 | ZNF200 | Homo sapiens zinc finger protein 200 (ZNF200), transcript variant 2, mRNA. (from RefSeq NM_198088) |
| chr16 | 3242027 | 3256627 | MEFV | Homo sapiens Mediterranean fever (MEFV), transcript variant 1, mRNA. (from RefSeq NM_000243) |
| chr16 | 3263742 | 3267567 | LINC00921 | Homo sapiens long intergenic non-protein coding RNA 921 (LINC00921), long non-coding RNA. (from RefSeq NR_033904) |
| chr16 | 3273608 | 3275807 | AJ003147.11 | Homo sapiens cDNA FLJ45461 fis, clone BRSTN2011899. (from mRNA AK127383) |
| chr16 | 3282942 | 3291460 | ZNF263 | Homo sapiens zinc finger protein 263 (ZNF263), mRNA. (from RefSeq NM_005741) |
| chr16 | 3292878 | 3293403 | LA16c-360H6.1 | LA16c-360H6.1 (from geneSymbol) |
| chr16 | 3298831 | 3305645 | TIGD7 | Homo sapiens tigger transposable element derived 7 (TIGD7), mRNA. (from RefSeq NM_033208) |
| chr16 | 3305485 | 3318852 | ZNF75A | Homo sapiens zinc finger protein 75a (ZNF75A), transcript variant 2, mRNA. (from RefSeq NM_153028) |
| chr16 | 3307572 | 3308393 | LA16c-360H6.3 | Homo sapiens piRNA piR-31115, complete sequence. (from mRNA DQ571003) |
| chr16 | 3355888 | 3357294 | OR2C1 | Homo sapiens olfactory receptor family 2 subfamily C member 1 (OR2C1), mRNA. (from RefSeq NM_012368) |
| chr16 | 3370978 | 3372740 | MTRNR2L4 | Homo sapiens MT-RNR2-like 4 (MTRNR2L4), mRNA. (from RefSeq NM_001190476) |
| chr16 | 3382080 | 3401065 | ZSCAN32 | Homo sapiens zinc finger and SCAN domain containing 32 (ZSCAN32), transcript variant 1, mRNA. (from RefSeq NM_001284527) |
| chr16 | 3382112 | 3397745 | LA16c-306E5.2 | Homo sapiens cervical cancer suppressor gene 5 mRNA, complete cds. (from mRNA AF390550) |
| chr16 | 3401419 | 3409370 | ZNF174 | Homo sapiens zinc finger protein 174 (ZNF174), transcript variant 1, mRNA. (from RefSeq NM_003450) |
| chr16 | 3432421 | 3443542 | ZNF597 | Homo sapiens zinc finger protein 597 (ZNF597), mRNA. (from RefSeq NM_152457) |
| chr16 | 3443610 | 3486963 | NAA60 | Homo sapiens N(alpha)-acetyltransferase 60, NatF catalytic subunit (NAA60), transcript variant 1, mRNA. (from RefSeq NM_001083601) |
| chr16 | 3458070 | 3515564 | LA16c-306E5.3 | Synthetic construct Homo sapiens gateway clone IMAGE:100018224 5' read CLUAP1 mRNA. (from mRNA CU677823) |
| chr16 | 3485380 | 3485469 | MIR6126 | Homo sapiens microRNA 6126 (MIR6126), microRNA. (from RefSeq NR_106741) |
| chr16 | 3493483 | 3495424 | C16orf90 | Homo sapiens chromosome 16 open reading frame 90 (C16orf90), mRNA. (from RefSeq NM_001080524) |
| chr16 | 3500923 | 3539048 | CLUAP1 | Homo sapiens clusterin associated protein 1 (CLUAP1), transcript variant 1, mRNA. (from RefSeq NM_015041) |
| chr16 | 3533428 | 3534258 | LA16c-390H2.1 | Homo sapiens cDNA clone IMAGE:40030343. (from mRNA BC113975) |
| chr16 | 3539032 | 3577391 | NLRC3 | Homo sapiens NLR family, CARD domain containing 3 (NLRC3), transcript variant 1, mRNA. (from RefSeq NM_178844) |
| chr16 | 3542738 | 3545513 | LA16c-390H2.4 | Sequence 193498 from Patent EP1572962. (from mRNA JD212474) |
| chr16 | 3581180 | 3583266 | RP11-461A8.1 | RP11-461A8.1 (from geneSymbol) |
| chr16 | 3581180 | 3611598 | SLX4 | Homo sapiens SLX4 structure-specific endonuclease subunit (SLX4), mRNA. (from RefSeq NM_032444) |
| chr16 | 3642937 | 3665472 | DNASE1 | Among other functions, seems to be involved in cell death by apoptosis. Binds specifically to G-actin and blocks actin polymerization (By similarity). (from UniProt P24855) |
| chr16 | 3650635 | 3651703 | RP11-461A8.4 | Homo sapiens cDNA clone IMAGE:5557641, partial cds. (from mRNA BC035759) |
| chr16 | 3658036 | 3717597 | TRAP1 | Homo sapiens TNF receptor-associated protein 1 (TRAP1), transcript variant 1, mRNA. (from RefSeq NM_016292) |
| chr16 | 3686997 | 3687380 | RP11-461A8.5 | RP11-461A8.5 (from geneSymbol) |
| chr16 | 3725053 | 3880726 | CREBBP | Homo sapiens CREB binding protein (CREBBP), transcript variant 1, mRNA. (from RefSeq NM_004380) |
| chr16 | 3931216 | 3946305 | RP11-462G12.2 | RP11-462G12.2 (from geneSymbol) |
| chr16 | 3947608 | 3950444 | RP11-462G12.1 | Homo sapiens uncharacterized LOC102724927 (LOC102724927), long non-coding RNA. (from RefSeq NR_120311) |
| chr16 | 3962650 | 4116185 | ADCY9 | Homo sapiens adenylate cyclase 9 (ADCY9), mRNA. (from RefSeq NM_001116) |
| chr16 | 4032011 | 4032936 | RP11-462G12.4 | RP11-462G12.4 (from geneSymbol) |
| chr16 | 4180116 | 4183515 | RP11-95P2.3 | RP11-95P2.3 (from geneSymbol) |
| chr16 | 4189373 | 4242080 | SRL | Homo sapiens sarcalumenin (SRL), mRNA. (from RefSeq NM_001098814) |
| chr16 | 4245824 | 4253789 | LINC01569 | Homo sapiens long intergenic non-protein coding RNA 1569 (LINC01569), long non-coding RNA. (from RefSeq NR_039999) |
| chr16 | 4257185 | 4273075 | TFAP4 | Homo sapiens transcription factor AP-4 (activating enhancer binding protein 4) (TFAP4), mRNA. (from RefSeq NM_003223) |
| chr16 | 4314760 | 4339597 | GLIS2 | Can act either as a transcriptional repressor or as a transcriptional activator, depending on the cell context. Acts as a repressor of the Hedgehog signaling pathway (By similarity). Represses the Hedgehog-dependent expression of Wnt4 (By similarity). Necessary to maintain the differentiated epithelial phenotype in renal cells through the inhibition of SNAI1, which itself induces the epithelial-to-mesenchymal transition (By similarity). Represses transcriptional activation mediated by CTNNB1 in the Wnt signaling pathway. May act by recruiting the corepressors CTBP1 and HDAC3. May be involved in neuron differentiation (By similarity). (from UniProt Q9BZE0) |
| chr16 | 4324666 | 4328340 | GLIS2-AS1 | Homo sapiens GLIS2 antisense RNA 1 (GLIS2-AS1), long non-coding RNA. (from RefSeq NR_110901) |
| chr16 | 4335869 | 4337818 | RP11-295D4.1 | Homo sapiens, clone IMAGE:4215072, mRNA. (from mRNA BC013195) |
| chr16 | 4340250 | 4416587 | CORO7-PAM16 | Homo sapiens CORO7-PAM16 readthrough (CORO7-PAM16), mRNA. (from RefSeq NM_001201479) |
| chr16 | 4340250 | 4355607 | PAM16 | Regulates ATP-dependent protein translocation into the mitochondrial matrix. Inhibits DNAJC19 stimulation of HSPA9/Mortalin ATPase activity. (from UniProt Q9Y3D7) |
| chr16 | 4346693 | 4348648 | RP11-295D4.3 | Homo sapiens cDNA clone IMAGE:3505922, partial cds. (from mRNA BC006120) |
| chr16 | 4354541 | 4416664 | CORO7 | Homo sapiens coronin 7 (CORO7), transcript variant 1, mRNA. (from RefSeq NM_024535) |
| chr16 | 4371847 | 4383528 | VASN | Homo sapiens vasorin (VASN), mRNA. (from RefSeq NM_138440) |
| chr16 | 4425804 | 4456775 | DNAJA3 | Homo sapiens DnaJ heat shock protein family (Hsp40) member A3 (DNAJA3), transcript variant 1, mRNA. (from RefSeq NM_005147) |
| chr16 | 4426901 | 4427380 | RP11-295D4.4 | Sequence 444112 from Patent EP1572962. (from mRNA JD463088) |
| chr16 | 4430521 | 4431103 | RP11-295D4.5 | RP11-295D4.5 (from geneSymbol) |
| chr16 | 4461690 | 4476306 | NMRAL1 | Redox sensor protein. Undergoes restructuring and subcellular redistribution in response to changes in intracellular NADPH/NADP(+) levels. At low NADPH concentrations the protein is found mainly as a monomer, and binds argininosuccinate synthase (ASS1), the enzyme involved in nitric oxide synthesis. Association with ASS1 impairs its activity and reduces the production of nitric oxide, which subsecuently prevents apoptosis. Under normal NADPH concentrations, the protein is found as a dimer and hides the binding site for ASS1. The homodimer binds one molecule of NADPH. Has higher affinity for NADPH than for NADP(+). Binding to NADPH is necessary to form a stable dimer. (from UniProt Q9HBL8) |
| chr16 | 4474702 | 4510346 | HMOX2 | Homo sapiens heme oxygenase 2 (HMOX2), transcript variant 4, mRNA. (from RefSeq NM_001127206) |
| chr16 | 4510674 | 4538470 | CDIP1 | Homo sapiens cell death-inducing p53 target 1 (CDIP1), transcript variant 1, mRNA. (from RefSeq NM_001199054) |
| chr16 | 4532215 | 4533670 | RP11-709D24.8 | RP11-709D24.8 (from geneSymbol) |
| chr16 | 4556489 | 4600714 | C16orf96 | Homo sapiens chromosome 16 open reading frame 96 (C16orf96), mRNA. (from RefSeq NM_001145011) |
| chr16 | 4560000 | 4561662 | RP11-709D24.5 | Sequence 411571 from Patent EP1572962. (from mRNA JD430547) |
| chr16 | 4608882 | 4614926 | UBALD1 | Homo sapiens UBA like domain containing 1 (UBALD1), mRNA. (from RefSeq NM_145253) |
| chr16 | 4624789 | 4690974 | MGRN1 | Homo sapiens mahogunin ring finger 1, E3 ubiquitin protein ligase (MGRN1), transcript variant 1, mRNA. (from RefSeq NM_015246) |
| chr16 | 4633590 | 4633881 | RN7SL850P | RNA, 7SL, cytoplasmic 850, pseudogene (from HGNC RN7SL850P) |
| chr16 | 4634328 | 4640623 | RP11-709D24.6 | Homo sapiens cDNA FLJ36026 fis, clone TESTI2016758. (from mRNA AK093345) |
| chr16 | 4658850 | 4659151 | Metazoa_SRP | Sequence 468519 from Patent EP1572962. (from mRNA JD487495) |
| chr16 | 4671317 | 4671390 | MIR6769A | Homo sapiens microRNA 6769a (MIR6769A), microRNA. (from RefSeq NR_106827) |
| chr16 | 4693693 | 4695859 | NUDT16L1 | Homo sapiens nudix hydrolase 16 like 1 (NUDT16L1), transcript variant 1, mRNA. (from RefSeq NM_032349) |
| chr16 | 4696511 | 4734162 | ANKS3 | Homo sapiens ankyrin repeat and sterile alpha motif domain containing 3 (ANKS3), transcript variant 1, mRNA. (from RefSeq NM_133450) |
| chr16 | 4730114 | 4752565 | RP11-127I20.7 | Homo sapiens cDNA FLJ50081 complete cds, highly similar to Zinc finger protein 500. (from mRNA AK297993) |
| chr16 | 4734271 | 4749396 | C16orf71 | Homo sapiens chromosome 16 open reading frame 71 (C16orf71), mRNA. (from RefSeq NM_139170) |
| chr16 | 4748238 | 4767218 | ZNF500 | Homo sapiens zinc finger protein 500 (ZNF500), transcript variant 1, mRNA. (from RefSeq NM_021646) |
| chr16 | 4756337 | 4756633 | Metazoa_SRP | Sequence 70392 from Patent EP1572962. (from mRNA JD089368) |
| chr16 | 4777668 | 4788521 | SEPT12 | Homo sapiens septin 12 (SEPT12), transcript variant 2, mRNA. (from RefSeq NM_144605) |
| chr16 | 4788396 | 4796491 | SMIM22 | Homo sapiens small integral membrane protein 22 (SMIM22), transcript variant 2, mRNA. (from RefSeq NM_001253791) |
| chr16 | 4795264 | 4796532 | RP11-127I20.5 | RP11-127I20.5 (from geneSymbol) |
| chr16 | 4796967 | 4802950 | ROGDI | Homo sapiens rogdi homolog (ROGDI), transcript variant 1, mRNA. (from RefSeq NM_024589) |
| chr16 | 4803202 | 4847342 | GLYR1 | Homo sapiens glyoxylate reductase 1 homolog (Arabidopsis) (GLYR1), transcript variant 1, mRNA. (from RefSeq NM_032569) |
| chr16 | 4839243 | 4840334 | RP11-127I20.8 | Sequence 478668 from Patent EP1572962. (from mRNA JD497644) |
| chr16 | 4847630 | 4882360 | UBN1 | Homo sapiens ubinuclein 1 (UBN1), transcript variant 2, mRNA. (from RefSeq NM_001079514) |
| chr16 | 4882506 | 4937135 | PPL | Homo sapiens periplakin (PPL), mRNA. (from RefSeq NM_002705) |
| chr16 | 4951772 | 4951891 | Y_RNA | Y_RNA (from geneSymbol) |
| chr16 | 4958316 | 5019158 | SEC14L5 | Homo sapiens SEC14 like lipid binding 5 (SEC14L5), mRNA. (from RefSeq NM_014692) |
| chr16 | 5024843 | 5033934 | NAGPA | Homo sapiens N-acetylglucosamine-1-phosphodiester alpha-N-acetylglucosaminidase (NAGPA), mRNA. (from RefSeq NM_016256) |
| chr16 | 5033701 | 5043999 | RP11-165E7.1 | Homo sapiens cDNA FLJ30660 fis, clone DFNES2000457. (from mRNA AK055222) |
| chr16 | 5044121 | 5066110 | C16orf89 | Homo sapiens chromosome 16 open reading frame 89 (C16orf89), transcript variant 1, mRNA. (from RefSeq NM_152459) |
| chr16 | 5071818 | 5085587 | ALG1 | Homo sapiens ALG1, chitobiosyldiphosphodolichol beta-mannosyltransferase (ALG1), mRNA. (from RefSeq NM_019109) |
| chr16 | 5084303 | 5097808 | EEF2KMT | Homo sapiens eukaryotic elongation factor 2 lysine methyltransferase (EEF2KMT), transcript variant 1, mRNA. (from RefSeq NM_201400) |
| chr16 | 5098738 | 5142595 | RP11-10K17.6 | RP11-10K17.6 (from geneSymbol) |
| chr16 | 5215393 | 5220594 | RP11-382N13.2 | RP11-382N13.2 (from geneSymbol) |
| chr16 | 5239801 | 6776014 | RP11-420N3.3 | Homo sapiens cDNA clone IMAGE:5244947, **** WARNING: chimeric clone ****. (from mRNA BC108660) |
| chr16 | 5239814 | 5600151 | RP11-420N3.2 | Homo sapiens cDNA clone IMAGE:5244947, **** WARNING: chimeric clone ****. (from mRNA BC108660) |
| chr16 | 5263222 | 5263371 | AC074051.1 | AC074051.1 (from geneSymbol) |
| chr16 | 5369820 | 5369915 | AC074051.2 | Sequence 136615 from Patent EP1572962. (from mRNA JD155591) |
| chr16 | 5601168 | 5616196 | LINC01570 | Homo sapiens long intergenic non-protein coding RNA 1570 (LINC01570), long non-coding RNA. (from RefSeq NR_110902) |
| chr16 | 5632466 | 5632566 | MIR8065 | Homo sapiens microRNA 8065 (MIR8065), microRNA. (from RefSeq NR_107032) |
| chr16 | 5760686 | 5760788 | AC012175.1 | Sequence 5130 from Patent WO2014113089. (from mRNA JC514877) |
| chr16 | 6037209 | 6038977 | RP11-509E10.3 | Sequence 470271 from Patent EP1572962. (from mRNA JD489247) |
| chr16 | 6056974 | 6092954 | RP11-509E10.1 | RP11-509E10.1 (from geneSymbol) |
| chr16 | 6573766 | 6577359 | RP11-468I15.1 | RP11-468I15.1 (from geneSymbol) |
| chr16 | 6703882 | 6705558 | RP11-185J20.1 | Homo sapiens pRbBP-39 mRNA, complete cds. (from mRNA AF204269) |
| chr16 | 6748907 | 6748969 | RNU7-99P | RNA, U7 small nuclear 99 pseudogene (from HGNC RNU7-99P) |
| chr16 | 6873898 | 6874005 | RNU6-457P | RNA, U6 small nuclear 457, pseudogene (from HGNC RNU6-457P) |
| chr16 | 7004006 | 7004112 | RNU6-328P | RNA, U6 small nuclear 328, pseudogene (from HGNC RNU6-328P) |
| chr16 | 7332749 | 7712504 | RBFOX1 | Homo sapiens RNA binding protein, fox-1 homolog (C. elegans) 1 (RBFOX1), transcript variant 1, mRNA. (from RefSeq NM_145891) |
| chr16 | 7614229 | 7614992 | RP11-26O3.1 | RP11-26O3.1 (from geneSymbol) |
| chr16 | 7726840 | 7726951 | SNORA40 | small nucleolar RNA, H/ACA box 40 (from HGNC SNORA40) |
| chr16 | 7888488 | 7894395 | CTD-2535I10.1 | CTD-2535I10.1 (from geneSymbol) |
| chr16 | 7899337 | 7899431 | AC093515.1 | Sequence 341184 from Patent EP1572962. (from mRNA JD360160) |
| chr16 | 8276262 | 8295700 | RP11-279O17.2 | RP11-279O17.2 (from geneSymbol) |
| chr16 | 8298491 | 8299772 | RP11-279O17.1 | Homo sapiens mRNA; cDNA DKFZp434F0535 (from clone DKFZp434F0535). (from mRNA AL117506) |
| chr16 | 8309538 | 8309657 | AC018767.1 | Sequence 136615 from Patent EP1572962. (from mRNA JD155591) |
| chr16 | 8309961 | 8357860 | RP11-279O17.3 | RP11-279O17.3 (from geneSymbol) |
| chr16 | 8369863 | 8376276 | RP11-568A19.1 | RP11-568A19.1 (from geneSymbol) |
| chr16 | 8526548 | 8532013 | RP11-483K5.3 | RP11-483K5.3 (from geneSymbol) |
| chr16 | 8569499 | 8589838 | TMEM114 | Homo sapiens transmembrane protein 114 (TMEM114), transcript variant 1, mRNA. (from RefSeq NM_001146336) |
| chr16 | 8621687 | 8649654 | METTL22 | Homo sapiens methyltransferase like 22 (METTL22), transcript variant 1, mRNA. (from RefSeq NM_024109) |
| chr16 | 8666761 | 8667271 | RP11-475D10.4 | Homo sapiens cDNA FLJ27474 fis, clone DMC07009. (from mRNA AK130984) |
| chr16 | 8674564 | 8784575 | ABAT | Homo sapiens 4-aminobutyrate aminotransferase (ABAT), transcript variant 1, mRNA. (from RefSeq NM_020686) |
| chr16 | 8683253 | 8683551 | RN7SL743P | RNA, 7SL, cytoplasmic 743, pseudogene (from HGNC RN7SL743P) |
| chr16 | 8683460 | 8683543 | AC007224.1 | AC007224.1 (from geneSymbol) |
| chr16 | 8705455 | 8705517 | RNU7-63P | RNA, U7 small nuclear 63 pseudogene (from HGNC RNU7-63P) |
| chr16 | 8795179 | 8797648 | TMEM186 | Homo sapiens transmembrane protein 186 (TMEM186), mRNA. (from RefSeq NM_015421) |
| chr16 | 8797816 | 8849331 | PMM2 | Homo sapiens phosphomannomutase 2 (PMM2), mRNA. (from RefSeq NM_000303) |
| chr16 | 8847649 | 8848724 | RP11-152P23.2 | RP11-152P23.2 (from geneSymbol) |
| chr16 | 8848104 | 8860417 | RP11-77H9.2 | RP11-77H9.2 (from geneSymbol) |
| chr16 | 8852942 | 8869012 | CARHSP1 | Homo sapiens calcium regulated heat stable protein 1 (CARHSP1), transcript variant 1, mRNA. (from RefSeq NM_014316) |
| chr16 | 8853311 | 8854347 | RP11-77H9.5 | RP11-77H9.5 (from geneSymbol) |
| chr16 | 8869250 | 8870032 | RP11-77H9.6 | RP11-77H9.6 (from geneSymbol) |
| chr16 | 8892093 | 8963484 | USP7 | Homo sapiens ubiquitin specific peptidase 7 (herpes virus-associated) (USP7), transcript variant 1, mRNA. (from RefSeq NM_003470) |
| chr16 | 8962705 | 8966990 | RP11-77H9.8 | RP11-77H9.8 (from geneSymbol) |
| chr16 | 9068553 | 9072412 | RP11-473I1.6 | RP11-473I1.6 (from geneSymbol) |
| chr16 | 9091647 | 9121640 | C16orf72 | Homo sapiens chromosome 16 open reading frame 72 (C16orf72), mRNA. (from RefSeq NM_014117) |
| chr16 | 9104847 | 9113181 | RP11-473I1.9 | Homo sapiens cDNA FLJ41568 fis, clone CTONG2003094. (from mRNA AK123562) |
| chr16 | 9105833 | 9107174 | RP11-473I1.5 | RP11-473I1.5 (from geneSymbol) |
| chr16 | 9355587 | 9408093 | RP11-243A14.1 | Homo sapiens mRNA; cDNA DKFZp686F1792 (from clone DKFZp686F1792). (from mRNA CR749525) |
| chr16 | 9441293 | 9444985 | LINC01177 | Homo sapiens long intergenic non-protein coding RNA 1177 (LINC01177), long non-coding RNA. (from RefSeq NR_126397) |
| chr16 | 9446009 | 9455505 | LINC01195 | Homo sapiens long intergenic non-protein coding RNA 1195 (LINC01195), long non-coding RNA. (from RefSeq NR_126349) |
| chr16 | 9466654 | 9517564 | RP11-418I22.2 | RP11-418I22.2 (from geneSymbol) |
| chr16 | 9542044 | 9614894 | RP11-418I22.3 | RP11-418I22.3 (from geneSymbol) |
| chr16 | 9564389 | 9564497 | RNA5SP403 | RNA, 5S ribosomal pseudogene 403 (from HGNC RNA5SP403) |
| chr16 | 9600096 | 9600228 | RNA5SP404 | RNA, 5S ribosomal pseudogene 404 (from HGNC RNA5SP404) |
| chr16 | 9666884 | 9676843 | RP11-297M9.1 | RP11-297M9.1 (from geneSymbol) |
| chr16 | 9753403 | 10182754 | GRIN2A | Homo sapiens glutamate ionotropic receptor NMDA type subunit 2A (GRIN2A), transcript variant 2, mRNA. (from RefSeq NM_000833) |
| chr16 | 9955460 | 9955563 | AC026423.1 | Sequence 149178 from Patent EP1572962. (from mRNA JD168154) |
| chr16 | 10033683 | 10037297 | RP11-895K13.2 | RP11-895K13.2 (from geneSymbol) |
| chr16 | 10221774 | 10222073 | RN7SL493P | RNA, 7SL, cytoplasmic 493, pseudogene (from HGNC RN7SL493P) |
| chr16 | 10351439 | 10352752 | RP11-609N14.1 | RP11-609N14.1 (from geneSymbol) |
| chr16 | 10386054 | 10483638 | ATF7IP2 | Homo sapiens activating transcription factor 7 interacting protein 2 (ATF7IP2), transcript variant 1, mRNA. (from RefSeq NM_024997) |
| chr16 | 10445308 | 10449747 | RP11-609N14.4 | Homo sapiens cDNA FLJ46216 fis, clone TESTI4012956. (from mRNA AK128095) |
| chr16 | 10496271 | 10496374 | RNU6-633P | RNA, U6 small nuclear 633, pseudogene (from HGNC RNU6-633P) |
| chr16 | 10514841 | 10528202 | LINC01290 | long intergenic non-protein coding RNA 1290 (from HGNC LINC01290) |
| chr16 | 10528421 | 10580698 | EMP2 | Homo sapiens epithelial membrane protein 2 (EMP2), mRNA. (from RefSeq NM_001424) |
| chr16 | 10529439 | 10532082 | RP11-27M24.1 | RP11-27M24.1 (from geneSymbol) |
| chr16 | 10576498 | 10578183 | RP11-27M24.2 | RP11-27M24.2 (from geneSymbol) |
| chr16 | 10627500 | 10694945 | TEKT5 | Homo sapiens tektin 5 (TEKT5), mRNA. (from RefSeq NM_144674) |
| chr16 | 10691272 | 10692973 | RP11-109M19.1 | RP11-109M19.1 (from geneSymbol) |
| chr16 | 10743844 | 10769351 | NUBP1 | Homo sapiens nucleotide binding protein 1 (NUBP1), transcript variant 1, mRNA. (from RefSeq NM_002484) |
| chr16 | 10761225 | 10818600 | TVP23A | Membrane; Multi-pass membrane protein (from UniProt A6NH52) |
| chr16 | 10864913 | 10888752 | RP11-876N24.2 | RP11-876N24.2 (from geneSymbol) |
| chr16 | 10877197 | 10932281 | CIITA | Homo sapiens class II, major histocompatibility complex, transactivator (CIITA), transcript variant 2, mRNA. (from RefSeq NM_000246) |
| chr16 | 10928890 | 10942460 | DEXI | Homo sapiens Dexi homolog (mouse) (DEXI), mRNA. (from RefSeq NM_014015) |
| chr16 | 10933902 | 10936280 | RP11-876N24.3 | Sequence 274535 from Patent EP1572962. (from mRNA JD293511) |
| chr16 | 10934257 | 10934887 | RP11-876N24.7 | RP11-876N24.7 (from geneSymbol) |
| chr16 | 10938885 | 10940044 | RP11-876N24.5 | Homo sapiens, clone IMAGE:4872114, mRNA. (from mRNA BC021142) |
| chr16 | 10940718 | 10943021 | RP11-876N24.4 | Homo sapiens cDNA FLJ30967 fis, clone HEART2000309, weakly similar to PTB-ASSOCIATED SPLICING FACTOR. (from mRNA AK055529) |
| chr16 | 10944487 | 11182189 | CLEC16A | Homo sapiens C-type lectin domain family 16 member A (CLEC16A), transcript variant 1, mRNA. (from RefSeq NM_015226) |
| chr16 | 11056555 | 11057034 | RP11-66H6.4 | RP11-66H6.4 (from geneSymbol) |
| chr16 | 11066495 | 11071102 | RP11-66H6.3 | RP11-66H6.3 (from geneSymbol) |
| chr16 | 11196176 | 11224969 | RP11-396B14.2 | RP11-396B14.2 (from geneSymbol) |
| chr16 | 11249648 | 11351755 | RMI2 | Essential component of the RMI complex, a complex that plays an important role in the processing of homologous recombination intermediates to limit DNA crossover formation in cells. The complex is therefore essential for the stability, localization, and function of complexes containing BLM. In the RMI complex, it is required to target BLM to chromatin and stress- induced nuclear foci and mitotic phosphorylation of BLM. (from UniProt Q96E14) |
| chr16 | 11254404 | 11256179 | SOCS1 | Homo sapiens suppressor of cytokine signaling 1 (SOCS1), mRNA. (from RefSeq NM_003745) |
| chr16 | 11267856 | 11269332 | TNP2 | Homo sapiens transition protein 2 (during histone to protamine replacement) (TNP2), mRNA. (from RefSeq NM_005425) |
| chr16 | 11273217 | 11273641 | PRM3 | Homo sapiens protamine 3 (PRM3), mRNA. (from RefSeq NM_021247) |
| chr16 | 11274112 | 11274236 | SNORA48 | small nucleolar RNA, H/ACA box 48 (from HGNC SNORA48) |
| chr16 | 11275638 | 11276480 | PRM2 | Homo sapiens protamine 2 (PRM2), transcript variant 1, mRNA. (from RefSeq NM_002762) |
| chr16 | 11280835 | 11281350 | PRM1 | Homo sapiens protamine 1 (PRM1), mRNA. (from RefSeq NM_002761) |
| chr16 | 11306439 | 11306527 | MIR548H2 | Homo sapiens microRNA 548 h-2 (MIR548H2), microRNA. (from RefSeq NR_031678) |
| chr16 | 11315066 | 11315178 | Y_RNA | Y_RNA (from geneSymbol) |
| chr16 | 11341808 | 11345211 | RP11-485G7.5 | RP11-485G7.5 (from geneSymbol) |
| chr16 | 11345482 | 11345560 | AC009121.2 | AC009121.2 (from geneSymbol) |
| chr16 | 11348142 | 11349321 | RP11-485G7.6 | Homo sapiens, clone IMAGE:5418366, mRNA. (from mRNA BC038217) |
| chr16 | 11348726 | 11348796 | AC009121.1 | AC009121.1 (from geneSymbol) |
| chr16 | 11372014 | 11523588 | CTD-3088G3.8 | The sequence shown here is derived from an Ensembl automatic analysis pipeline and should be considered as preliminary data. (from UniProt M0QZD8) |
| chr16 | 11465259 | 11473174 | CTD-3088G3.6 | Homo sapiens uncharacterized LOC101927131 (LOC101927131), long non-coding RNA. (from RefSeq NR_110907) |
| chr16 | 11549612 | 11629210 | LITAF | Probable role in regulating transcription of specific genes. May regulate through NFKB1 the expression of the CCL2/MCP-1 chemokine. May play a role in tumor necrosis factor alpha (TNF- alpha) gene expression. (from UniProt Q99732) |
| chr16 | 11668413 | 11679159 | SNN | Homo sapiens stannin (SNN), mRNA. (from RefSeq NM_003498) |
| chr16 | 11679079 | 11742878 | TXNDC11 | Homo sapiens thioredoxin domain containing 11 (TXNDC11), transcript variant 2, mRNA. (from RefSeq NM_015914) |
| chr16 | 11741909 | 11744506 | RP11-490O6.2 | Sequence 204985 from Patent EP1572962. (from mRNA JD223961) |
| chr16 | 11750585 | 11797267 | ZC3H7A | Homo sapiens zinc finger CCCH-type containing 7A (ZC3H7A), mRNA. (from RefSeq NM_014153) |
| chr16 | 11753665 | 11753948 | Metazoa_SRP | Sequence 175658 from Patent EP1572962. (from mRNA JD194634) |
| chr16 | 11797467 | 11798275 | RP11-486I11.2 | RP11-486I11.2 (from geneSymbol) |
| chr16 | 11819849 | 11828811 | BCAR4 | Homo sapiens breast cancer anti-estrogen resistance 4 (non-protein coding) (BCAR4), transcript variant 1, long non-coding RNA. (from RefSeq NR_024049) |
| chr16 | 11833849 | 11851585 | RSL1D1 | Homo sapiens ribosomal L1 domain containing 1 (RSL1D1), mRNA. (from RefSeq NM_015659) |
| chr16 | 11851648 | 11895611 | RP11-166B2.8 | RP11-166B2.8 (from geneSymbol) |
| chr16 | 11868127 | 11915920 | GSPT1 | Homo sapiens G1 to S phase transition 1 (GSPT1), transcript variant 1, mRNA. (from RefSeq NM_002094) |
| chr16 | 11881074 | 11882569 | RP11-166B2.3 | Sequence 146476 from Patent EP1572962. (from mRNA JD165452) |
| chr16 | 11908207 | 11908916 | RP11-166B2.5 | RP11-166B2.5 (from geneSymbol) |
| chr16 | 11915660 | 11915738 | AC007216.1 | Sequence 192291 from Patent EP1572962. (from mRNA JD211267) |
| chr16 | 11927372 | 11942045 | RP11-166B2.1 | Nucleus (from UniProt A6NJ64) |
| chr16 | 11965106 | 11968068 | TNFRSF17 | Homo sapiens tumor necrosis factor receptor superfamily member 17 (TNFRSF17), mRNA. (from RefSeq NM_001192) |
| chr16 | 11976737 | 12574289 | SNX29 | Homo sapiens sorting nexin 29 (SNX29), mRNA. (from RefSeq NM_032167) |
| chr16 | 11976850 | 11977850 | RP11-166B2.7 | RP11-166B2.7 (from geneSymbol) |
| chr16 | 12086745 | 12090302 | RP11-276H1.3 | Homo sapiens cDNA FLJ32790 fis, clone TESTI2002361. (from mRNA AK057352) |
| chr16 | 12093626 | 12095307 | RP11-276H1.2 | RP11-276H1.2 (from geneSymbol) |
| chr16 | 12297196 | 12297323 | ACA64 | ACA64 (from geneSymbol) |
| chr16 | 12366981 | 12372582 | RP11-165M1.3 | RP11-165M1.3 (from geneSymbol) |
| chr16 | 12372822 | 12373897 | RP11-165M1.2 | RP11-165M1.2 (from geneSymbol) |
| chr16 | 12374354 | 12376032 | RP11-165M1.1 | Homo sapiens cDNA FLJ27129 fis, clone SPL07676. (from mRNA AK130639) |
| chr16 | 12545481 | 12546684 | RP11-552C15.1 | RP11-552C15.1 (from geneSymbol) |
| chr16 | 12556352 | 12557694 | CTD-3037G24.4 | CTD-3037G24.4 (from geneSymbol) |
| chr16 | 12560755 | 12611044 | CTD-3037G24.3 | CTD-3037G24.3 (from geneSymbol) |
| chr16 | 12614450 | 12614852 | CTD-3037G24.5 | CTD-3037G24.5 (from geneSymbol) |
| chr16 | 12659798 | 12804017 | CPPED1 | Homo sapiens calcineurin-like phosphoesterase domain containing 1 (CPPED1), transcript variant 1, mRNA. (from RefSeq NM_018340) |
| chr16 | 12720320 | 12720371 | MIR4718 | Homo sapiens microRNA 4718 (MIR4718), microRNA. (from RefSeq NR_039869) |
| chr16 | 12745872 | 12757835 | CTD-2583P5.1 | CTD-2583P5.1 (from geneSymbol) |
| chr16 | 12759281 | 12761162 | CTD-2583P5.3 | Homo sapiens, clone IMAGE:5551658, mRNA. (from mRNA BC035765) |
| chr16 | 12842486 | 12842610 | SNORA27 | small nucleolar RNA, H/ACA box 27 (from HGNC SNORA27) |
| chr16 | 12901619 | 13240413 | SHISA9 | Homo sapiens shisa family member 9 (SHISA9), transcript variant 1, mRNA. (from RefSeq NM_001145204) |
| chr16 | 13043471 | 13043576 | AC092380.1 | Sequence 14 from Patent EP2733219. (from mRNA JC505995) |
| chr16 | 13197605 | 13204907 | AC009134.1 | AC009134.1 (from geneSymbol) |
| chr16 | 13246315 | 13562918 | U91319.1 | U91319.1 (from geneSymbol) |
| chr16 | 13331367 | 13332583 | AC003009.1 | AC003009.1 (from geneSymbol) |
| chr16 | 13730136 | 13779748 | U95743.1 | U95743.1 (from geneSymbol) |
| chr16 | 13920156 | 13952345 | ERCC4 | Homo sapiens excision repair cross-complementation group 4 (ERCC4), mRNA. (from RefSeq NM_005236) |
| chr16 | 13930676 | 13935635 | CTD-2135D7.2 | CTD-2135D7.2 (from geneSymbol) |
| chr16 | 13953154 | 13954825 | CTD-2135D7.3 | CTD-2135D7.3 (from geneSymbol) |
| chr16 | 14009279 | 14016016 | CTD-2135D7.5 | Homo sapiens uncharacterized LOC101927311 (LOC101927311), long non-coding RNA. (from RefSeq NR_110909) |
| chr16 | 14018879 | 14021077 | CTD-2135D7.4 | Homo sapiens uncharacterized LOC101927348 (LOC101927348), long non-coding RNA. (from RefSeq NR_110915) |
| chr16 | 14071321 | 14266771 | MKL2 | Acts as a transcriptional coactivator of serum response factor (SRF). Required for skeletal myogenic differentiation. (from UniProt Q9ULH7) |
| chr16 | 14150832 | 14153235 | CTA-276F8.1 | CTA-276F8.1 (from geneSymbol) |
| chr16 | 14191819 | 14200277 | CTA-276F8.2 | CTA-276F8.2 (from geneSymbol) |
| chr16 | 14275507 | 14275600 | Y_RNA | Y_RNA (from geneSymbol) |
| chr16 | 14301388 | 14326744 | MIR193BHG | MIR193BHG (from geneSymbol) |
| chr16 | 14303966 | 14304049 | MIR193B | Homo sapiens microRNA 193b (MIR193B), microRNA. (from RefSeq NR_030177) |
| chr16 | 14309269 | 14309380 | MIR365A | microRNA 365a (from HGNC MIR365A) |
| chr16 | 14363108 | 14370266 | RP11-65J21.1 | Homo sapiens uncharacterized LOC105447648 (LOC105447648), long non-coding RNA. (from RefSeq NR_131191) |
| chr16 | 14408038 | 14418872 | RP11-65J21.4 | RP11-65J21.4 (from geneSymbol) |
| chr16 | 14435700 | 14630267 | PARN | Homo sapiens poly(A)-specific ribonuclease (PARN), transcript variant 1, mRNA. (from RefSeq NM_002582) |
| chr16 | 14601822 | 14602094 | RN7SL694P | RNA, 7SL, cytoplasmic 694, pseudogene (from HGNC RN7SL694P) |
| chr16 | 14632814 | 14669236 | BFAR | Homo sapiens bifunctional apoptosis regulator (BFAR), mRNA. (from RefSeq NM_016561) |
| chr16 | 14643787 | 14643849 | RNU7-125P | RNA, U7 small nuclear 125 pseudogene (from HGNC RNU7-125P) |
| chr16 | 14672544 | 14694669 | PLA2G10 | Homo sapiens phospholipase A2 group X (PLA2G10), transcript variant 1, mRNA. (from RefSeq NM_003561) |
| chr16 | 14695566 | 14707055 | RP11-82O18.2 | RP11-82O18.2 (from geneSymbol) |
| chr16 | 14711688 | 14726293 | NPIPA3 | Homo sapiens nuclear pore complex interacting protein family member A3 (NPIPA3), mRNA. (from RefSeq NM_001277323) |
| chr16 | 14734684 | 14746177 | RP11-719K4.6 | RP11-719K4.6 (from geneSymbol) |
| chr16 | 14750812 | 14765413 | NPIPA2 | Homo sapiens nuclear pore complex interacting protein family member A2 (NPIPA2), mRNA. (from RefSeq NM_001277324) |
| chr16 | 14833680 | 14896160 | NOMO1 | Homo sapiens NODAL modulator 1 (NOMO1), mRNA. (from RefSeq NM_014287) |
| chr16 | 14901498 | 14902174 | MIR3179-1 | microRNA 3179-1 (from HGNC MIR3179-1) |
| chr16 | 14901507 | 14901591 | MIR3179-1 | Homo sapiens microRNA 3179-1 (MIR3179-1), microRNA. (from RefSeq NR_036140) |
| chr16 | 14907716 | 14907781 | MIR3670-1 | Homo sapiens microRNA 3670-1 (MIR3670-1), microRNA. (from RefSeq NR_037442) |
| chr16 | 14909886 | 14911345 | MIR3180-1 | microRNA 3180-1 (from HGNC MIR3180-1) |
| chr16 | 14911219 | 14911313 | MIR3180-1 | Homo sapiens microRNA 3180-1 (MIR3180-1), microRNA. (from RefSeq NR_036141) |
| chr16 | 14915456 | 14915556 | Y_RNA | Y_RNA (from geneSymbol) |
| chr16 | 14925936 | 14926003 | MIR6511A1 | Homo sapiens microRNA 6511a-1 (MIR6511A1), microRNA. (from RefSeq NR_106766) |
| chr16 | 14930819 | 14930879 | MIR6770-1 | Homo sapiens microRNA 6770-1 (MIR6770-1), microRNA. (from RefSeq NR_106828) |
| chr16 | 14937442 | 14952056 | NPIPA1 | Homo sapiens nuclear pore complex interacting protein family member A1 (NPIPA1), mRNA. (from RefSeq NM_006985) |
| chr16 | 14975102 | 15038332 | PDXDC1 | Homo sapiens pyridoxal-dependent decarboxylase domain containing 1 (PDXDC1), transcript variant 1, mRNA. (from RefSeq NM_015027) |
| chr16 | 15010320 | 15010397 | MIR1972-1 | Homo sapiens microRNA 1972-1 (MIR1972-1), microRNA. (from RefSeq NR_036054) |
| chr16 | 15015827 | 15016390 | RP11-680G24.6 | RP11-680G24.6 (from geneSymbol) |
| chr16 | 15037852 | 15056064 | NTAN1 | Homo sapiens N-terminal asparagine amidase (NTAN1), transcript variant 1, mRNA. (from RefSeq NM_173474) |
| chr16 | 15060021 | 15094317 | RRN3 | Homo sapiens RRN3 homolog, RNA polymerase I transcription factor (RRN3), transcript variant 1, mRNA. (from RefSeq NM_018427) |
| chr16 | 15094410 | 15109197 | RP11-72I8.1 | Homo sapiens uncharacterized LOC100505915 (LOC100505915), long non-coding RNA. (from RefSeq NR_125434) |
| chr16 | 15104722 | 15131601 | PKD1P6 | Homo sapiens PKD1P6-NPIPP1 readthrough (PKD1P6-NPIPP1), transcript variant 2, non-coding RNA. (from RefSeq NR_123722) |
| chr16 | 15130077 | 15130137 | AC139256.1 | AC139256.1 (from geneSymbol) |
| chr16 | 15134065 | 15134150 | MIR6511B2 | Homo sapiens microRNA 6511b-2 (MIR6511B2), microRNA. (from RefSeq NR_106965) |
| chr16 | 15150696 | 15150796 | Y_RNA | Y_RNA (from geneSymbol) |
| chr16 | 15154849 | 15155002 | MIR3180-4 | Homo sapiens microRNA 3180-4 (MIR3180-4), microRNA. (from RefSeq NR_037466) |
| chr16 | 15154902 | 15157020 | MIR3180-4 | microRNA 3180-4 (from HGNC MIR3180-4) |
| chr16 | 15158456 | 15158521 | AC126763.1 | Sequence 3225 from Patent WO2014113089. (from mRNA JC512972) |
| chr16 | 15363623 | 15378294 | NPIPA5 | Homo sapiens nuclear pore complex interacting protein family member A5 (NPIPA5), mRNA. (from RefSeq NM_001277325) |
| chr16 | 15395753 | 15515348 | RP11-1021N1.1 | The sequence shown here is derived from an Ensembl automatic analysis pipeline and should be considered as preliminary data. (from UniProt H3BMD7) |
| chr16 | 15395778 | 15408439 | MPV17L | Homo sapiens MPV17 mitochondrial membrane protein-like (MPV17L), transcript variant 1, mRNA. (from RefSeq NM_001128423) |
| chr16 | 15434294 | 15588259 | C16orf45 | Homo sapiens chromosome 16 open reading frame 45 (C16orf45), transcript variant 1, mRNA. (from RefSeq NM_033201) |
| chr16 | 15594385 | 15643166 | KIAA0430 | Homo sapiens KIAA0430 (KIAA0430), transcript variant 1, mRNA. (from RefSeq NM_014647) |
| chr16 | 15608473 | 15610563 | CTB-193M12.1 | CTB-193M12.1 (from geneSymbol) |
| chr16 | 15611029 | 15611095 | MIR6506 | Homo sapiens microRNA 6506 (MIR6506), microRNA. (from RefSeq NR_106761) |
| chr16 | 15643266 | 15726353 | NDE1 | Homo sapiens nudE neurodevelopment protein 1 (NDE1), transcript variant 1, mRNA. (from RefSeq NM_001143979) |
| chr16 | 15643280 | 15643390 | MIR484 | microRNA 484 (from HGNC MIR484) |
| chr16 | 15683289 | 15684570 | CTB-193M12.3 | CTB-193M12.3 (from geneSymbol) |
| chr16 | 15701236 | 15702118 | CTB-193M12.5 | CTB-193M12.5 (from geneSymbol) |
| chr16 | 15703990 | 15857033 | MYH11 | Homo sapiens myosin, heavy chain 11, smooth muscle (MYH11), transcript variant SM1A, mRNA. (from RefSeq NM_002474) |
| chr16 | 15726673 | 15732993 | AF001548.5 | AF001548.5 (from geneSymbol) |
| chr16 | 15741150 | 15741791 | AF001548.6 | AF001548.6 (from geneSymbol) |
| chr16 | 15790974 | 15792717 | AF001548.3 | Homo sapiens cDNA FLJ26903 fis, clone RCT01362. (from mRNA AK130413) |
| chr16 | 15865719 | 15888615 | FOPNL | Homo sapiens FGFR1OP N-terminal like (FOPNL), transcript variant 1, mRNA. (from RefSeq NM_144600) |
| chr16 | 15877162 | 15877266 | RNU6-213P | RNA, U6 small nuclear 213, pseudogene (from HGNC RNU6-213P) |
| chr16 | 15885028 | 15886158 | CTA-972D3.2 | CTA-972D3.2 (from geneSymbol) |
| chr16 | 15949576 | 16143062 | ABCC1 | Homo sapiens ATP binding cassette subfamily C member 1 (ABCC1), mRNA. (from RefSeq NM_004996) |
| chr16 | 16148927 | 16223464 | ABCC6 | Isoform 1: May participate directly in the active transport of drugs into subcellular organelles or influence drug distribution indirectly. Transports glutathione conjugates as leukotriene-c4 (LTC4) and N-ethylmaleimide S-glutathione (NEM-GS). (from UniProt O95255) |
| chr16 | 16223026 | 16224261 | RP11-517A5.7 | RP11-517A5.7 (from geneSymbol) |
| chr16 | 16232494 | 16294814 | NOMO3 | Homo sapiens NODAL modulator 3 (NOMO3), mRNA. (from RefSeq NM_001004067) |
| chr16 | 16290134 | 16292242 | RP11-517A5.5 | RP11-517A5.5 (from geneSymbol) |
| chr16 | 16300158 | 16300242 | MIR3179-2 | Homo sapiens microRNA 3179-2 (MIR3179-2), microRNA. (from RefSeq NR_036143) |
| chr16 | 16306369 | 16306434 | MIR3670-2 | Homo sapiens microRNA 3670-2 (MIR3670-2), microRNA. (from RefSeq NR_049832) |
| chr16 | 16308541 | 16310000 | MIR3180-2 | microRNA 3180-2 (from HGNC MIR3180-2) |
| chr16 | 16309874 | 16309968 | MIR3180-2 | Homo sapiens microRNA 3180-2 (MIR3180-2), microRNA. (from RefSeq NR_036142) |
| chr16 | 16314111 | 16314211 | Y_RNA | Y_RNA (from geneSymbol) |
| chr16 | 16324587 | 16324654 | MIR6511A2 | Homo sapiens microRNA 6511a-2 (MIR6511A2), microRNA. (from RefSeq NR_106969) |
| chr16 | 16329304 | 16329364 | MIR6770-2 | Homo sapiens microRNA 6770-2 (MIR6770-2), microRNA. (from RefSeq NR_107060) |
| chr16 | 16331876 | 16350590 | AC138969.4 | Homo sapiens hypothetical protein LOC339047, mRNA (cDNA clone IMAGE:4179257), complete cds. (from mRNA BC046145) |
| chr16 | 16368875 | 16368942 | MIR6511A3 | Homo sapiens microRNA 6511a-3 (MIR6511A3), microRNA. (from RefSeq NR_106970) |
| chr16 | 16372603 | 16372663 | AC138969.1 | AC138969.1 (from geneSymbol) |
| chr16 | 16379054 | 16393954 | NPIPA7 | Homo sapiens nuclear pore complex interacting protein family member A7 (NPIPA7), mRNA. (from RefSeq NM_001282507) |
| chr16 | 16658226 | 16664135 | RP11-14N9.2 | RP11-14N9.2 (from geneSymbol) |
| chr16 | 16709401 | 16709528 | AC092326.1 | AC092326.1 (from geneSymbol) |
| chr16 | 16760904 | 16762465 | RP11-14N9.1 | RP11-14N9.1 (from geneSymbol) |
| chr16 | 17082294 | 17085049 | CTD-2576D5.1 | CTD-2576D5.1 (from geneSymbol) |
| chr16 | 17101768 | 17470881 | XYLT1 | Homo sapiens xylosyltransferase I (XYLT1), mRNA. (from RefSeq NM_022166) |
| chr16 | 17134503 | 17138736 | CTD-2576D5.4 | CTD-2576D5.4 (from geneSymbol) |
| chr16 | 17192862 | 17194232 | RP11-1102P22.3 | RP11-1102P22.3 (from geneSymbol) |
| chr16 | 17227022 | 17228000 | RP11-1102P22.1 | Homo sapiens cDNA: FLJ21731 fis, clone COLF1695. (from mRNA AK025384) |
| chr16 | 17249673 | 17251395 | RP11-1102P22.2 | Homo sapiens cDNA FLJ26221 fis, clone ADG08311. (from mRNA AK129732) |
| chr16 | 17445824 | 17446380 | RP11-567P19.1 | RP11-567P19.1 (from geneSymbol) |
| chr16 | 17540330 | 17540985 | RP11-916L7.1 | RP11-916L7.1 (from geneSymbol) |
| chr16 | 17825251 | 17826906 | CTD-3229J4.1 | CTD-3229J4.1 (from geneSymbol) |
| chr16 | 17961596 | 17962370 | CTA-481E9.2 | CTA-481E9.2 (from geneSymbol) |
| chr16 | 17967392 | 18151595 | CTA-481E9.4 | CTA-481E9.4 (from geneSymbol) |
| chr16 | 18002805 | 18173063 | CTA-481E9.3 | CTA-481E9.3 (from geneSymbol) |
| chr16 | 18201500 | 18201956 | CTD-2036A2.1 | Sequence 256474 from Patent EP1572962. (from mRNA JD275450) |
| chr16 | 18263635 | 18263756 | AC126755.3 | AC126755.3 (from geneSymbol) |
| chr16 | 18317941 | 18336736 | NPIPA8 | Belongs to the NPIP family. (from UniProt P0DM63) |
| chr16 | 18339243 | 18339303 | AC126755.1 | AC126755.1 (from geneSymbol) |
| chr16 | 18344012 | 18344079 | MIR6511A4 | Homo sapiens microRNA 6511a-4 (MIR6511A4), microRNA. (from RefSeq NR_106971) |
| chr16 | 18358094 | 18376839 | RP11-1212A22.4 | Homo sapiens hypothetical protein LOC339047, mRNA (cDNA clone IMAGE:4179257), complete cds. (from mRNA BC046145) |
| chr16 | 18379350 | 18379410 | MIR6770-3 | Homo sapiens microRNA 6770-3 (MIR6770-3), microRNA. (from RefSeq NR_107061) |
| chr16 | 18383229 | 18383314 | AC126755.2 | Sequence 5435 from Patent WO2014113089. (from mRNA JC515182) |
| chr16 | 18397936 | 18398036 | Y_RNA | Y_RNA (from geneSymbol) |
| chr16 | 18402145 | 18403604 | MIR3180-3 | microRNA 3180-3 (from HGNC MIR3180-3) |
| chr16 | 18402177 | 18402271 | MIR3180-3 | Homo sapiens microRNA 3180-3 (MIR3180-3), microRNA. (from RefSeq NR_036144) |
| chr16 | 18405697 | 18405762 | MIR3670-3 | Homo sapiens microRNA 3670-3 (MIR3670-3), microRNA. (from RefSeq NR_128712) |
| chr16 | 18411308 | 18411851 | MIR3179-3 | microRNA 3179-3 (from HGNC MIR3179-3) |
| chr16 | 18411893 | 18411977 | MIR3179-3 | Homo sapiens microRNA 3179-3 (MIR3179-3), microRNA. (from RefSeq NR_036145) |
| chr16 | 18488300 | 18488365 | MIR3670-4 | Homo sapiens microRNA 3670-4 (MIR3670-4), microRNA. (from RefSeq NR_128713) |
| chr16 | 18494492 | 18494576 | MIR3179-4 | Homo sapiens microRNA 3179-4 (MIR3179-4), microRNA. (from RefSeq NR_128709) |
| chr16 | 18499924 | 18562112 | NOMO2 | Homo sapiens NODAL modulator 2 (NOMO2), transcript variant 2, mRNA. (from RefSeq NM_173614) |
| chr16 | 18570454 | 18571683 | RP11-457I16.2 | RP11-457I16.2 (from geneSymbol) |
| chr16 | 18649698 | 18649825 | AC138811.1 | AC138811.1 (from geneSymbol) |
| chr16 | 18781294 | 18790334 | RPS15A | Homo sapiens ribosomal protein S15a (RPS15A), transcript variant 2, mRNA. (from RefSeq NM_001019) |
| chr16 | 18788062 | 18801519 | RP11-1035H13.3 | The sequence shown here is derived from an Ensembl automatic analysis pipeline and should be considered as preliminary data. (from UniProt H3BN98) |
| chr16 | 18791666 | 18801678 | ARL6IP1 | Homo sapiens ADP ribosylation factor like GTPase 6 interacting protein 1 (ARL6IP1), transcript variant 1, mRNA. (from RefSeq NM_015161) |
| chr16 | 18803082 | 18812181 | RP11-1035H13.2 | RP11-1035H13.2 (from geneSymbol) |
| chr16 | 18804852 | 18926454 | SMG1 | Homo sapiens SMG1 phosphatidylinositol 3-kinase-related kinase (SMG1), mRNA. (from RefSeq NM_015092) |
| chr16 | 18926862 | 18937043 | CTD-2288F12.1 | CTD-2288F12.1 (from geneSymbol) |
| chr16 | 18930195 | 18930282 | AC092287.1 | Sequence 3242 from Patent WO2014113089. (from mRNA JC512989) |
| chr16 | 18983933 | 19063181 | TMC7 | Homo sapiens transmembrane channel like 7 (TMC7), transcript variant 1, mRNA. (from RefSeq NM_024847) |
| chr16 | 19012156 | 19012263 | RNU6-1340P | RNA, U6 small nuclear 1340, pseudogene (from HGNC RNU6-1340P) |
| chr16 | 19062861 | 19067691 | RP11-626G11.5 | RP11-626G11.5 (from geneSymbol) |
| chr16 | 19065990 | 19066694 | RP11-626G11.1 | Homo sapiens full length insert cDNA clone YY74E10. (from mRNA AF088010) |
| chr16 | 19067598 | 19080095 | COQ7 | Homo sapiens coenzyme Q7 homolog, ubiquinone (yeast) (COQ7), transcript variant 1, mRNA. (from RefSeq NM_016138) |
| chr16 | 19086855 | 19098110 | RP11-626G11.4 | RP11-626G11.4 (from geneSymbol) |
| chr16 | 19111034 | 19111484 | RP11-626G11.6 | RP11-626G11.6 (from geneSymbol) |
| chr16 | 19114001 | 19173578 | CTD-2349B8.1 | The sequence shown here is derived from an Ensembl automatic analysis pipeline and should be considered as preliminary data. (from UniProt I3L2K1) |
| chr16 | 19114382 | 19121629 | ITPRIPL2 | Homo sapiens inositol 1,4,5-trisphosphate receptor interacting protein-like 2 (ITPRIPL2), transcript variant 1, mRNA. (from RefSeq NM_001034841) |
| chr16 | 19119975 | 19121629 | RP11-626G11.3 | RP11-626G11.3 (from geneSymbol) |
| chr16 | 19168248 | 19268334 | SYT17 | Homo sapiens synaptotagmin 17 (SYT17), transcript variant 1, mRNA. (from RefSeq NM_016524) |
| chr16 | 19285778 | 19304400 | CLEC19A | Secreted (from UniProt Q6UXS0) |
| chr16 | 19285782 | 19310946 | AC003003.5 | Homo sapiens mRNA; cDNA DKFZp686A05212 (from clone DKFZp686A05212). (from mRNA BX640722) |
| chr16 | 19315163 | 19316526 | CTA-363E6.1 | CTA-363E6.1 (from geneSymbol) |
| chr16 | 19343646 | 19401693 | CTA-363E6.2 | CTA-363E6.2 (from geneSymbol) |
| chr16 | 19392212 | 19393468 | CTA-363E6.3 | Homo sapiens, clone IMAGE:4616265, mRNA. (from mRNA BC020735) |
| chr16 | 19410728 | 19411662 | CTA-363E6.5 | CTA-363E6.5 (from geneSymbol) |
| chr16 | 19456449 | 19499113 | TMC5 | Homo sapiens transmembrane channel like 5 (TMC5), transcript variant 3, mRNA. (from RefSeq NM_024780) |
| chr16 | 19476915 | 19487899 | CTA-363E6.6 | CTA-363E6.6 (from geneSymbol) |
| chr16 | 19498609 | 19498747 | RNU4-46P | RNA, U4 small nuclear 46, pseudogene (from HGNC RNU4-46P) |
| chr16 | 19501688 | 19522145 | GDE1 | Homo sapiens glycerophosphodiester phosphodiesterase 1 (GDE1), mRNA. (from RefSeq NM_016641) |
| chr16 | 19501688 | 19502286 | CTA-363E6.7 | CTA-363E6.7 (from geneSymbol) |
| chr16 | 19503860 | 19504625 | CTA-363E6.8 | CTA-363E6.8 (from geneSymbol) |
| chr16 | 19523810 | 19553408 | CCP110 | Homo sapiens centriolar coiled-coil protein 110 kDa (CCP110), transcript variant 2, mRNA. (from RefSeq NM_014711) |
| chr16 | 19555719 | 19700689 | C16orf62 | Membrane; Single-pass membrane protein (from UniProt Q7Z3J2) |
| chr16 | 19694034 | 19694428 | AC002550.6 | AC002550.6 (from geneSymbol) |
| chr16 | 19701933 | 19718234 | KNOP1 | Homo sapiens lysine-rich nucleolar protein 1 (KNOP1), mRNA. (from RefSeq NM_001012991) |
| chr16 | 19706350 | 19715383 | AC002550.5 | AC002550.5 (from geneSymbol) |
| chr16 | 19716455 | 19858467 | IQCK | Homo sapiens IQ motif containing K (IQCK), transcript variant 1, mRNA. (from RefSeq NM_153208) |
| chr16 | 19761171 | 19766099 | CTD-2380F24.1 | CTD-2380F24.1 (from geneSymbol) |
| chr16 | 19856690 | 19884917 | GPRC5B | Homo sapiens G protein-coupled receptor class C group 5 member B (GPRC5B), transcript variant 1, mRNA. (from RefSeq NM_016235) |
| chr16 | 19890500 | 19890597 | AC134300.1 | AC134300.1 (from geneSymbol) |
| chr16 | 20031484 | 20073917 | GPR139 | Homo sapiens G protein-coupled receptor 139 (GPR139), transcript variant 1, mRNA. (from RefSeq NM_001002911) |
| chr16 | 20208924 | 20209525 | RP11-204E4.3 | RP11-204E4.3 (from geneSymbol) |
| chr16 | 20309571 | 20327580 | GP2 | Homo sapiens glycoprotein 2 (GP2), transcript variant 2, mRNA. (from RefSeq NM_001502) |
| chr16 | 20333068 | 20356301 | UMOD | Uromodulin: Functions in biogenesis and organization of the apical membrane of epithelial cells of the thick ascending limb of Henle's loop (TALH), where it promotes formation of complex filamentous gel-like structure providing the water barrier permeability. May serve as a receptor for binding and endocytosis for cytokines (IL-1, IL-2) and TNF. Facilitates neutrophil migration across renal epithelial. (from UniProt P07911) |
| chr16 | 20359169 | 20404737 | PDILT | Homo sapiens protein disulfide isomerase-like, testis expressed (PDILT), mRNA. (from RefSeq NM_174924) |
| chr16 | 20385956 | 20391164 | RP11-429K17.1 | RP11-429K17.1 (from geneSymbol) |
| chr16 | 20409533 | 20441336 | ACSM5 | Homo sapiens acyl-CoA synthetase medium-chain family member 5 (ACSM5), mRNA. (from RefSeq NM_017888) |
| chr16 | 20441249 | 20447000 | CTD-2194A8.2 | CTD-2194A8.2 (from geneSymbol) |
| chr16 | 20451575 | 20487667 | ACSM2A | Has medium-chain fatty acid:CoA ligase activity with broad substrate specificity (in vitro). Acts on acids from C(4) to C(11) and on the corresponding 3-hydroxy- and 2,3- or 3,4- unsaturated acids (in vitro) (By similarity). (from UniProt Q08AH3) |
| chr16 | 20487701 | 20489659 | CTD-3076M17.1 | Homo sapiens cDNA FLJ34659 fis, clone KIDNE2018863. (from mRNA AK091978) |
| chr16 | 20536225 | 20576367 | ACSM2B | Homo sapiens acyl-CoA synthetase medium-chain family member 2B (ACSM2B), transcript variant 2, mRNA. (from RefSeq NM_001105069) |
| chr16 | 20580998 | 20586640 | RP11-143N13.2 | RP11-143N13.2 (from geneSymbol) |
| chr16 | 20623485 | 20697744 | ACSM1 | Has medium-chain fatty acid:CoA ligase activity with broad substrate specificity (in vitro). Acts on acids from C(4) to C(11) and on the corresponding 3-hydroxy- and 2,3- or 3,4- unsaturated acids (in vitro). Functions as GTP-dependent lipoate- activating enzyme that generates the substrate for lipoyltransferase (By similarity). (from UniProt Q08AH1) |
| chr16 | 20733663 | 20742084 | THUMPD1 | Homo sapiens THUMP domain containing 1 (THUMPD1), transcript variant 1, mRNA. (from RefSeq NM_017736) |
| chr16 | 20743662 | 20766620 | AC004381.7 | AC004381.7 (from geneSymbol) |
| chr16 | 20763701 | 20797581 | ACSM3 | Homo sapiens acyl-CoA synthetase medium-chain family member 3 (ACSM3), transcript variant 1, mRNA. (from RefSeq NM_005622) |
| chr16 | 20784508 | 20784615 | RNU6-944P | RNA, U6 small nuclear 944, pseudogene (from HGNC RNU6-944P) |
| chr16 | 20796343 | 20806473 | ERI2 | Homo sapiens ERI1 exoribonuclease family member 2 (ERI2), transcript variant 1, mRNA. (from RefSeq NM_001142725) |
| chr16 | 20806498 | 20849668 | AC004381.6 | Homo sapiens exonuclease NEF-sp (LOC81691), transcript variant 1, mRNA. (from RefSeq NM_030941) |
| chr16 | 20823442 | 20823537 | U4atac | U4atac (from geneSymbol) |
| chr16 | 20854924 | 20900384 | DCUN1D3 | Homo sapiens defective in cullin neddylation 1 domain containing 3 (DCUN1D3), mRNA. (from RefSeq NM_173475) |
| chr16 | 20900234 | 20925004 | LYRM1 | May promote cell proliferation and inhibition of apoptosis of preadipocytes. (from UniProt O43325) |
| chr16 | 20933110 | 21159441 | DNAH3 | Homo sapiens dynein axonemal heavy chain 3 (DNAH3), mRNA. (from RefSeq NM_017539) |
